# Root transcriptome of two contrasting *indica* rice cultivars uncovers regulators of root development and physiological responses

**DOI:** 10.1038/srep39266

**Published:** 2016-12-21

**Authors:** Alka Singh, Pramod Kumar, Vibhav Gautam, Balakrishnan Rengasamy, Bijan Adhikari, Makarla Udayakumar, Ananda K. Sarkar

**Affiliations:** 1 National Institute of Plant Genome Research, Aruna Asaf Ali Marg, New Delhi 110067, India; 2 Rice Research Station, Chinsurah, West Bengal 712101, India; 3 Department of Crop Physiology, University of Agricultural Sciences, GKVK, Bangalore 560065, India

## Abstract

The huge variation in root system architecture (RSA) among different rice (*Oryza sativa*) cultivars is conferred by their genetic makeup and different growth or climatic conditions. Unlike model plant *Arabidopsis*, the molecular basis of such variation in RSA is very poorly understood in rice. Cultivars with stable variation are valuable resources for identification of genes involved in RSA and related physiological traits. We have screened for RSA and identified two such *indica* rice cultivars, IR-64 (OsAS83) and IET-16348 (OsAS84), with stable contrasting RSA. OsAS84 produces robust RSA with more crown roots, lateral roots and root hairs than OsAS83. Using comparative root transcriptome analysis of these cultivars, we identified genes related to root development and different physiological responses like abiotic stress responses, hormone signaling, and nutrient acquisition or transport. The two cultivars differ in their response to salinity/dehydration stresses, phosphate/nitrogen deficiency, and different phytohormones. Differential expression of genes involved in salinity or dehydration response, nitrogen (N) transport, phosphate (Pi) starvation signaling, hormone signaling and root development underlies more resistance of OsAS84 towards abiotic stresses, Pi or N deficiency and its robust RSA. Thus our study uncovers gene-network involved in root development and abiotic stress responses in rice.

A wide range of variations, in terms of yield, physiology and morphology including root system architecture (RSA) trait are observed among various cultivars of rice (*Oryza sativa*)^1^. *O. sativa* has two major varieties - the upland varieties, usually with small number of deeper and thicker roots and cultivated in dry fields of temperate East Asia, and lowland varieties, usually with higher number of shallow and thin roots with low root/shoot mass ratio for better adaptation to submergence and cultivated in submerged fields of tropical Asia^2^. RSA of different cultivars vary tremendously to meet the requirement of the plant to combat various growth conditions such as nutrient deficiency, drought or sanity stress. Some rice cultivars are tolerant to abiotic stresses; Nagina 22 (mutant NH219) is drought tolerant, Pokkali is salinity tolerant and Dular is low phosphate tolerant^3^,^4^. The variation in RSA is regulated by intrinsic factors, such as transcription factors, microRNAs and phytohormones, and influenced by several extrinsic factors (like light and water), and a cumulative effect of all these leads to development of a particular type of RSA^5^.

In monocotyledonous plants, RSA consists of primary root (PR), lateral roots (LRs), crown/seminal roots (CR) and root hairs (RHs)^5^,^6^. Root is developed from root stem cells, which are present in the root apical meristem (RAM)^7^. Different genes and transcription factors, such as *PLETHORA (PLT*), *SHORT ROOT (SHR*), *SCARECROW (SCR*) and *WUSCHEL RELATED HOMEOBOX5 (WOX5*) regulate the maintenance of root meristem and its pattern in *Arabidopsis*^8^. In rice, homologs of these genes have also been identified; *OsWOX11* regulates CR emergence, *OsWOX3A* regulates LR development and RH formation, and *OsSCR1* and *OsSCR2* are involved in regulation of asymmetric division of cortex/endodermis progenitor cells and initiation of root development^9^,^10^,^11^.

Phytohormones such as auxin and cytokinin have been implicated in regulation of various RSA traits^12^. In rice, auxin and AUX/IAA (AUXIN/INDOLE ACETIC ACID) proteins regulate the expression of *CROWN ROOTLESS1 (CRL1)*, which encodes for *Arabidopsis* homologs LBD16/LBD19, which are ASYMMETRIC LEAVES (AS2)/LATERAL ORGAN BOUNDARIES (LOB) family transcription factor^13^. *crl1* mutant showed defects in CR formation and alteration in other auxin-related RSA traits such as reduced LR number, auxin insensitivity in LR formation and impaired root gravitropism^13^. The *CRL1* promoter contains an auxin responsive element (ARE), which binds with the OsARF16, a ortholog of ARF7 and ARF19 of *Arabidopsis*^14^. ARF7 and ARF19 regulates the expression of *LBD16* and *LBD29* and control lateral organ development^15^. ARF7 and ARF19 also show close relationship to CRL1 in phylogenetic study^16^,^17^. Knockdown of *OsARF16* resulted in reduced sensitivity of PR, LRs and RHs towards auxin and phosphate (Pi) deficiency^18^. Mutation in *OsIAA23*, another auxin responsive gene required for quiescent center (QC) maintenance, led to pleiotropic defects in root cap, LR and CR development^19^.

The major or minor nutrients also influence various aspects of root development, such as root length, diameter, LR branching, root angle, and other physiological changes^20^. Pi deficiency leads to reduced PR length and increased LR density in *Arabidopsis* with altered hormone sensitivity^21^,^22^,^23^. In rice, Pi deficiency leads to increased PR length^24^,^25^. Pi deficiency leads to redistribution of auxin content^26^,^27^. Low Pi condition for several days resulted in irreversible inhibition of root growth in *Arabidopsis*^28^. Availability of Nitrogen (N) has drastic and contrasting effects on development of RSA in different species or varieties. In *Arabidopsis*, both PR and LRs elongation is reduced on increased nitrate availability, whereas in rice and maize, PR gets elongated in N deprivation^29^,^30^.

Besides nutrient availability, soil composition and environmental stresses such as drought, high-salt and freezing factors also affect root development. Drought is one of the major constraints to many crop plants, especially to rice yields^1^. Under water deficiency, growth of leaves and stem is restricted, but root may continue to elongate^31^. Till date, many stress-related genes have been shown to play role in drought tolerance. Root specific overexpression of *OsNAC10* and *OsNAC5* resulted in enhanced root growth and increased drought tolerance^32^,^33^. In *Arabidopsis*, overexpression of a member of aspartic protease gene family, *ASPARTIC PROTEASE IN GUARD CELL 1(ASPG1*) leads to the reduction in water loss in ABA-dependent manner^34^.

Recent advances in various emerging phenomics tools, invasive or non-invasive imaging techniques and next generation sequencing (NGS) are helpful in correlating the genetic signature with RSA trait in rice^35^,^36^. Despite several efforts, our knowledge of regulation of monocot root development is still inadequate. Therefore dissecting the molecular and genetic regulatory mechanisms involved in root development is a prerequisite for the development of new rice varieties with improved RSA traits^37^. To uncover the molecular basis of RSA trait in rice, we have screened two dozen rice varieties and identified two *indica* rice cultivars, IR-64 and IET-16348 (OsAS83, lowland and OsAS84, upland, respectively) with stable and contrasting RSA trait as a model system. In this study, we have compared the root transcriptome of these cultivars and identified genes potentially involved in root development and related physiological responses. Our results indicate that differential expression of various transporters, transcription factors and genes involved in hormonal signaling could largely contribute to the phenotypic and physiological differences observed between two cultivars.

## Results and Discussion

### The OsAS83 and OsAS84 rice cultivars differ in their RSA

**Figure 1:**
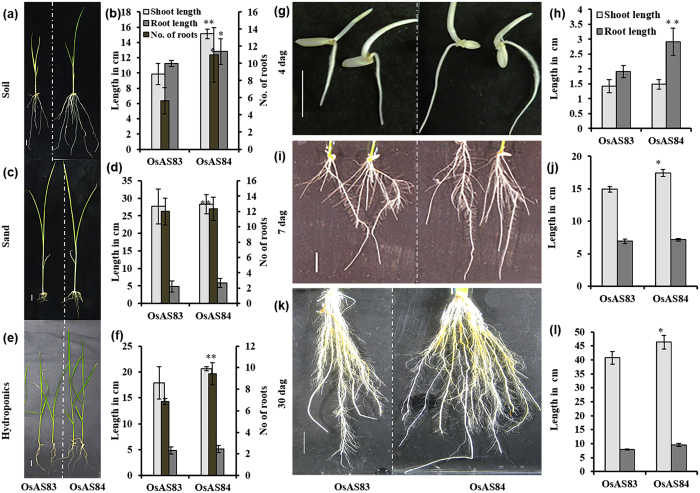
OsAS84 has advanced root system architecture (RSA) than OsAS83. (**a**–**f**) Phenotypic difference in RSA of OsAS83 and OsAS84 rice cultivars at 14 days after germination (dag). (**a**,**c** and **e**) Phenotypic difference between two cultivars in soil, sand and hydroponics, respectively. (**b**,**d** and **f**) Difference in primary root length, shoot length and number of crown roots in two cultivars in soil, sand and hydroponics, respectively. (**g**–**l**) Difference in RSA of OsAS83 and OsAS84 rice cultivars at different developmental stages in hydroponics. (**g**,**i** and **k**) Phenotypic difference of RSA between two cultivars at 4 dag, 7 dag and 30 dag, respectively. (**h**,**j** and **l**) Difference in primary root length and shoot length at 4 dag, 7 dag and 30 dag, respectively in two cultivars. Scale bar 1 cm. Error bars indicate standard error (n = 10). Asterisks indicate significant statistical differences, ***P < 0.001, **P < 0.01, *P < 0.05 (One-way ANOVA). Experiment was repeated three times with reproducible results.

It has been reported earlier that root system is highly plastic and vary drastically in terms of RSA in different growth conditions^5^. We screened two dozen of selected *indica* rice cultivars in different growth conditions for detailed analysis of their RSA (Fig. S1). In different growth conditions (soil, sand and hydroponics), OsAS83 and OsAS84 cultivars maintained stable and contrasting root growth pattern and RSA at 14 days after germination (dag). In all the conditions tested, OsAS84 showed more robust RSA than OsAS83. In soil, OsAS84 showed 11% longer PR and two-times more CR number in comparison to OsAS83 (Fig. 1a and b). In sand, the PR length was found to be significantly affected in both the cultivars and difference between PR length and CR number between two cultivars was reduced in comparison to soil (Fig. 1c and d). In hydroponics system, OsAS84 showed 33% higher number of CRs in comparison to OsAS83 (Fig. 1e,f and Fig. S2). We performed growth assay at different developmental stages and found that OsAS84 has longer PR and more number of CRs than OsAS83 at 4 dag, 7 dag and 30 dag (Fig. 1g–l). Interestingly, shoot of OsAS84 was also found to be longer than OsAS83 at 4 dag, 7 dag and 30 dag.

To check the histological variation between these two cultivars, we analyzed the transverse sections of 2 dag PR tip (at 0–1 cm). The epidermal and cortical cell layer was more compact in OsAS84 than OsAS83. OsAS84 also showed higher number of cortical cell layers in comparison to OsAS83, although there is no difference in vascular patterning (Fig. S3a and 3b). RH density was also found to be more in OsAS84 (Fig. S3c). For survival in dry temperate regions, the upland rice varieties tends to develop thicker and bushy roots and plants exposed to salinity and drought stress developed deeper roots with more branches^2^,^38^. Our analysis showed that OsAS84, an upland cultivar, produces longer PR, more CR, LR and RH density in different growing mediums as well as in developmental stages in comparison to OsAS83, a lowland cultivar. These advanced RSA traits of OsAS84 is indicative of its better adaptability against stress.

### OsAS83 and OsAS84 differ in shoot, flowering and seed traits

**Figure 2:**
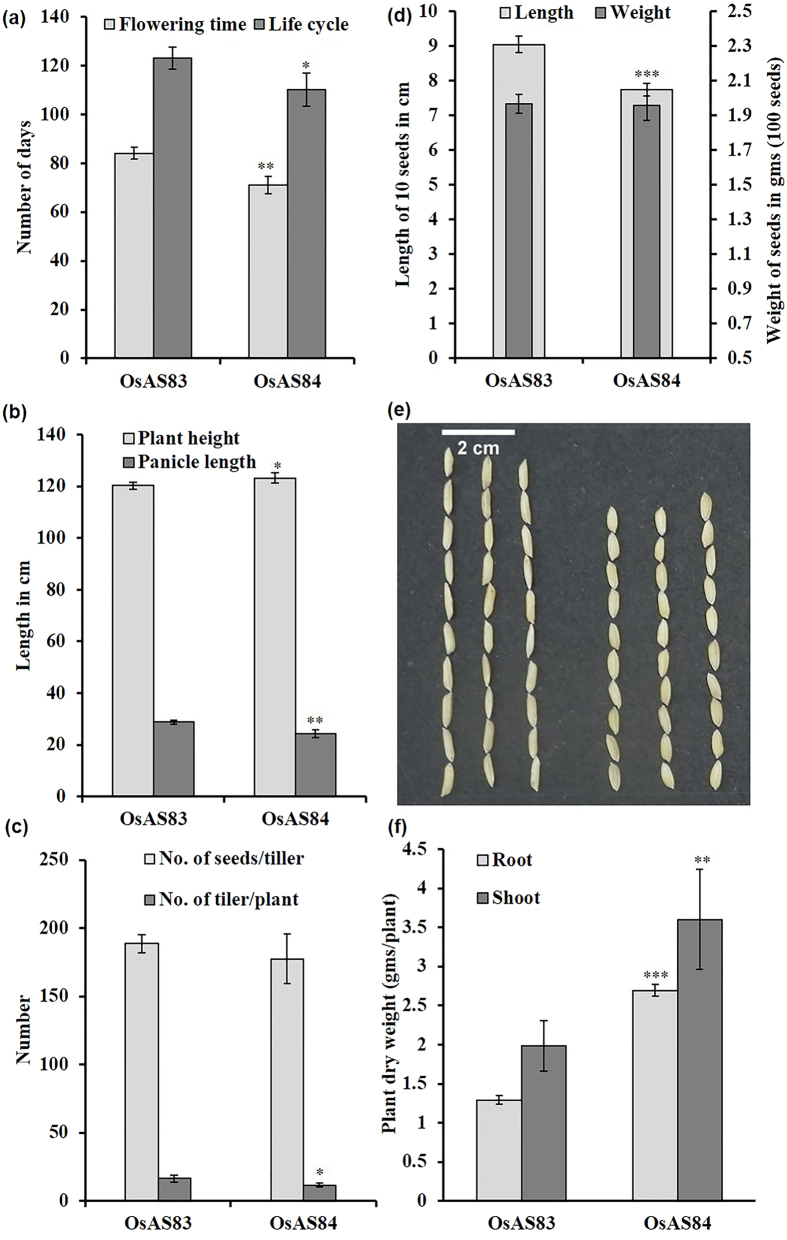
OsAS84 and OsAs83 differ in various agronomic traits. (**a**) OsAS84 has shorter flowering time than OsAS83. Error bars indicate SD (n = 10). (**b**) OsAS84 have longer plant height, and lower panicle length than OsAS83. (**c**) Number of seeds/tiller, and number of tillers/plant was lower in OsAS84. Error bars indicate SD (n = 10). (**d**) and (**e**) OsAS84 has smaller seed length and equal seed weight as OsAS83. Scale bar is 1 cm. (**f**), OsAS84 have more root and shoot dry weight than OsAS83. Shorter life-span of OsAS84 does not hamper the seed-vigour and final productivity. Asterisks indicate significant statistical differences, ***P < 0.001, **P < 0.01, *P < 0.05 (One-way ANOVA). Each experiment was repeated three times with similar results. Error bars indicate SD (n = 10).

To understand the effect of differential RSA traits on overall plant growth various phenotypic traits were analyzed. OsAS84 has smaller life-cycle (10.5% less) as well as early flowering time (by 10 ± 2 days), in comparison to OsAS83 (Fig. 2a). Height of fully mature plant show slight variation (120 ± 5 cm and 123 ± 5 cm for OsAS83 and OsAS84, respectively). It is possible that advanced RSA trait helped OsAS84 to grow more efficiently in less time (Fig. 2b). Average tiller number/plant and seeds number/tiller were reduced in OsAS84 by ~6% and ~30%, respectively (Fig. 2c). Grain size of OsAS84 was found to be 1% shorter in length and thicker in diameter in relation to OsAS83 seeds. Weight of seeds (per 100 seeds) showed no difference between two cultivars (Fig. 2d and e). Dry weight of both root and shoot was found to be more in OsAS84 (Fig. 2f). Therefore, despite having advanced RSA, there was no yield penalty in OsAS84.

### Differential root transcriptome signature underlies variation in RSA of OsAS83 and OsAS84

**Figure 3:**
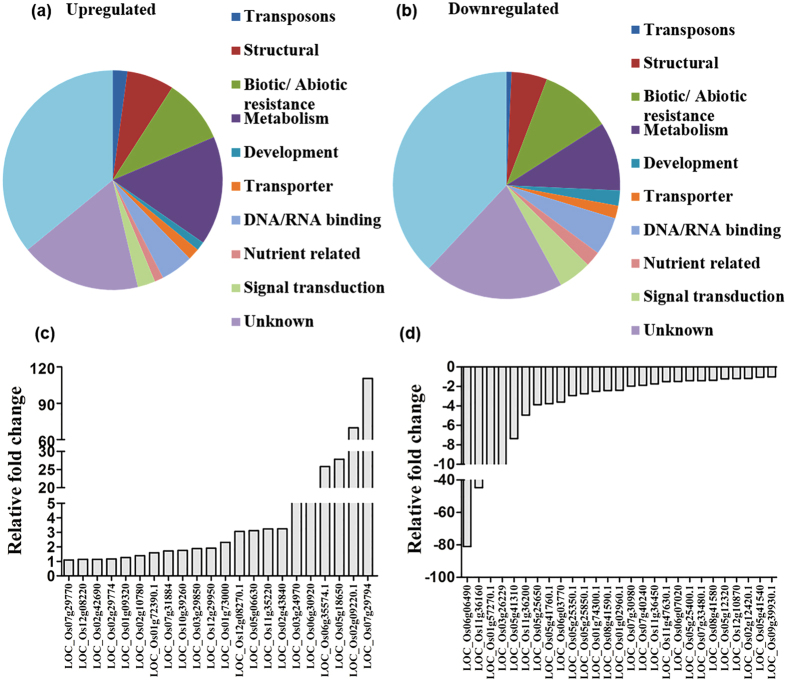
Comparative root transcriptome of OsAS83 and OsAS84 identifies genes of developmental and physiological importance. Microarray between 14 dag roots of two cultivars showed differential expression of several transporters, transcription factors and dehydration responsive genes. (**a**,**b**) Upregulated genes (231) and downregulated genes (276) in OsAS84 were classified according to their functional annotation. (**c**,**d**) Some of differentially expressed genes were further validated by real time qRT-PCR. All are statistically significant, P < 0.05 (n = 3).

Analysis of variation in gene expression pattern between rice cultivars with stable contrasting RSA is likely to uncovers molecular factors responsible for RSA trait. To identify the differentially expressed genes between the root tissues (at 14 dag) of OsAS83 and OsAS84 cultivars, we performed gene expression microarray. After taking a cut-off of P ≤ 0.05 and fold change ≥2.0, a total of 231 genes were found to be upregulated and 276 genes were found to be downregulated in OsAS84 root in comparison to OsAS83. We performed Gene Ontology (GO) analysis to annotate the function of differentially expressed genes which were categorized in 11 classes, for e.g. transposon, structural, biotic/abiotic resistance, metabolism, developmental, transporter, DNA/RNA binding, nutrient related, signal transduction, unknown and unidentified (Fig. 3a and b). Microarray results were further validated for some of the selected genes by real time quantitative RT-PCR (qRT-PCR) analysis (Fig. 3c,d and Table S1).

### Several transporters are differentially expressed in the roots of OsAS83 and OsAS84

We identified that several genes encoding for transporters are differentially expressed in roots of two cultivars. We observed that the metal cation transporter (LOC_Os03g29850.1), transmembrane amino acid transporter (LOC_Os05g14820.1), major facilitator transporter (LOC_Os02g02170.1), nitrate chloride transporter (LOC_Os12g29950.1), sugar transporter MtN3 (LOC_Os05g12320.1) and ABC transporter (LOC_Os06g03770.1) were differentially expressed in roots of these two cultivars (Table 1). Homologs of LOC_Os03g29850 (a metal cation transporter) and LOC_Os07g31884 (MATE efflux family protein) proteins in *Arabidopsis* are known to maintain Zinc homeostasis^39^. MATE transporter family is a large family with 58 orthologs in *Arabidopsis* and 40 members in rice^40^. In *Arabidopsis*, members of MATE transporters are known to be involved in pathogen infection, aluminum (Al) tolerance, sequestration of proanthocyanids, and glucose assimilation^41^,^42^,^43^,^44^. MATE transporter from sorghum, *Hordeum, Triticum* and maize have also been shown to participate in Al tolerance by forming non-toxic complexes of citrate with Al in soil solution^45^,^46^,^47^,^48^. Overexpression of *OsMATE1* and *OsMATE2* in *Arabidopsis* leads to altered development and pathogen susceptibility^49^. Overexpression of *AtATM3*, a homolog of ABC transporter (LOC_Os06g03770) showed enhanced Cd(II) and Pb(II) resistance in *Arabidopsis*^50^. Overexpression of *AtATM3* in *Brassica juncea* also conferred increased tolerance to Cd(II) and Pb(II) stresses^51^. Higher expression of MATE and other transporters indicate possible ameliorate adaptation of OsAS84 towards metal toxicity, which need to be tested further, and also reflects the conservation of this mechanism in rice.

For adapting to various growth conditions like presence of heavy metals, availability of nutrients in the soil, and abiotic stresses, RSA has to undergo developmental changes which is regulated by various molecular regulators. These developmental changes may often involve changes in the distribution and transport of sugars in shoot and root. In our transcriptome study, the expression of a sugar transporter (MtN3; SWEET gene family member) was downregulated in OsAS84 root (Fig. 3d). SWEET proteins are uniporters, which facilitate diffusion of sugars across cell membranes, and loading of sucrose into phloem^52^. Mutant of a member of *SWEET* gene family in *Arabidopsis, atsweet11/12* exhibited reduced root length upon germination on sugar-free media^53^. In rice, *OsSWEET11* and *OsSWEET14* have been reported to regulate rice reproductive development^54^,^55^. Expression of a nitrogen chloride transporter (*OsNCT*; LOC_Os12g29950.1) and *OsSPX2* (LOC_Os02g10780) was higher in OsAS84. The homolog of a nitrate chloride transporter, major facilitator protein (AT2G39210.1), was found to be upregulated in salt tolerant halophyte salt cress in comparison to *Arabidopsis*, which suggests its involvement in salinity stress^56^. Differential expression of these transporters in OsAS83 and OsAS84 roots suggests that these two cultivars might differ in their ability for nutrition acquisition or heavy metal toxicity tolerance or stress response, which further may lead to developmental variation in RSA. Although a matter of further study, the expression level of these transporters possibly link the developmental adaptability of RSA and physiological responses of plants.

### OsAS84 shows better ion absorption efficiency

**Figure 4:**
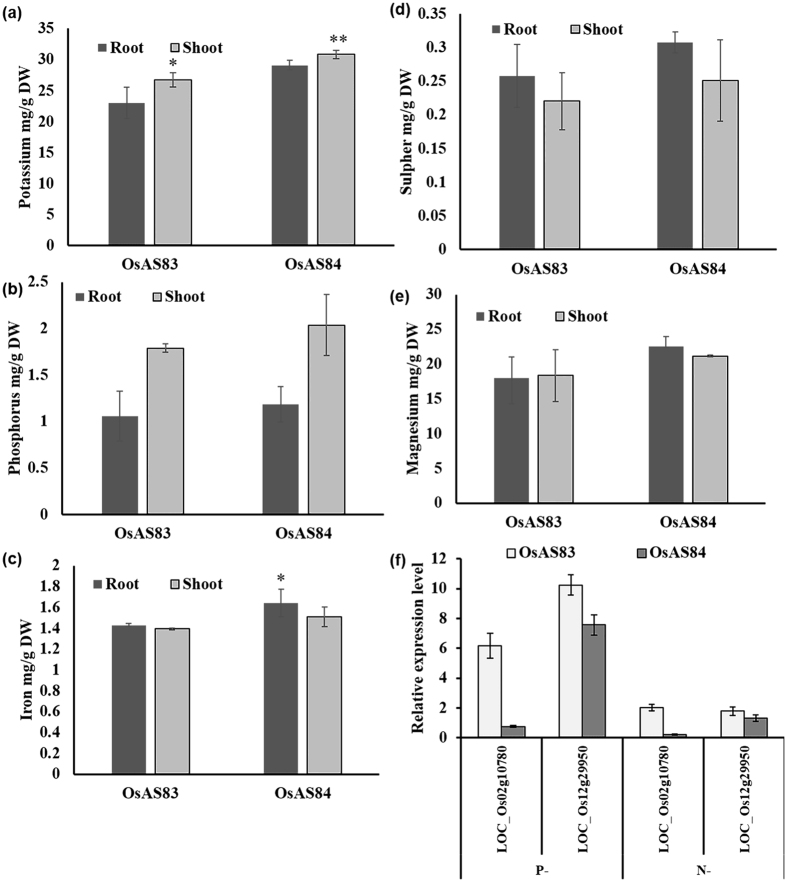
OsAS84 shows better ion absorption efficiency. (**a**–**e**) Concentration of K, P, Fe, S and Mg in root and shoot of OsAS83 and OsAS84. Error bars indicate SE (n = 3). (**f**) Expression of *OsSPX2* (LOC_Os02g10780) and nitrate chloride transporter (LOC_Os12g29950) in Pi deficiency/N deficiency (Pi−/N−) conditions. For ΔΔCt study Pi+/N+ conditions were used as control. Error bars indicate SE (n = 3). Asterisks indicate significant statistical differences, ***P < 0.001, **P < 0.01, *P < 0.05 (One-way ANOVA).

Differential expression of transporters may lead to differential acquisition of concerned micro/macro nutrients by roots. The concentration of various elements was measured in both roots and shoots of OsAS83 and OsAS84, which showed contrasting variation. We observed that the concentration of K, P, Fe, S, and Mg ions was more in both root and shoot tissue of OsAS84 than in OsAS83 (Fig. 4a–e). Higher concentration of various micro/macro elements in OsAS84 suggests that, this cultivar with advanced RSA has better ion absorption efficiency than OsAS83. More surface area of absorption (more root branches and RHs) and higher expression of several transporters (as indicated above) in OsAS84 roots could contribute to its enhanced ability of ion uptake. In same line, we have studied the expression of *OsNCT* and *OsSPX2* in N and Pi deficit conditions, respectively. In N or Pi deprived conditions, the expression of these transporters were induced in both the cultivars in comparison to N or Pi sufficient conditions (Fig. 4f). Thus, some RSA traits and expression level of transporters may be associated with ion transport efficiency in rice cultivars.

### Differential expression of hormone signaling genes is associated with RSA trait variation, in OsAS83 and OsAS84

Many genes involved in hormone signaling, are also involved in root development and in regulation of nutrient deficiency responses^57^. Hormone related genes like, ethylene responsive gene (LOC_Os02g43840), gibberellic acid stimulated transcript (GAST) (LOC_Os11g13670), and *OsIAA20* (LOC_Os06g07040, an Auxin-responsive Aux/IAA gene family member) were differentially expressed in roots of two cultivars (Table 1). Similar to *INDUCED BY PHOSPHATE STARVATION 1 (IPS1)* of *Arabidopsis*, LOC_Os02g43840 of rice was also shown to have ability to modulate the activity of rice *miR399*, which is a key regulator of phosphate signaling^58^. Involvement of Ethylene Response Factors (ERFs) in Pi starvation was also supported by the observation that five members of ERFs showed upregulation in Pi deficient conditions^59^. Differential expression of LOC_Os02g43840 (a rice ERF having the potential to sequester to *miR399*) and LOC_Os02g10780 (*OsSPX2,* a key member of phosphate starvation-induced signaling), further strengthen the hypothesis that these two cultivar differs in their responses to Pi deficiency. Members of GA responsive, GAST gene family in rice and maize were shown to be expressed in RAM and in mutant defective in root branching, indicating their role in root development^60^. Role of Aux/IAA proteins in root development have been described previously^13^. In maize, a member of Aux/IAA domain containing protein, *RUM1 (ROOTLESS WITH UNDETECTABLE MERISTEM 1*) was shown to regulate crown and seminal root development^61^. Mutation in *Arabidopsis IAA13* (At2g33310), a homolog of rice LOC_Os06g07040, resulted in seedlings with no root, due to failure of specification and abnormal cell division in the embryonic root meristem^62^. Differential accumulation of these genes in two cultivars indicates probable role of these genes in RSA regulation.

Recently it has been reported that overexpression of *CYP71Z2* in rice leads to resistance to *Xanthomonas oryzae* and in reduction of *OsIAA20* transcript level, indicating involvement of auxin signaling in disease resistance, besides its role in various aspects of plant development^63^. Majority of rice resistance (R) genes, which confer resistance to *X. oryzae*, belong to Nucleotide-Binding Site Leucine-Rich Repeat (NBS-LRR) or LRR Kinase super families^64^,^65^. In our microarray data, the expression of LOC_Os11g39190.1 and LOC_Os11g14110.1, two NBS-LRR domain containing proteins coding genes, were found to be upregulated in OsAS84 roots. Another member of cytochrome P450 family, *CYP87A3* was found to be down regulated in OsAS84. This protein functions as a negative regulator for the auxin responsiveness of growth^66^. Accumulation of R genes, suppression of *OsIAA20* and *CYP* genes, suggests that plant defense pathway and root developmental pathway share some common signaling mechanism, including hormone signaling.

### Transcription factors involved in various signaling pathways are differentially expressed in OsAS83 and OsAS84 roots

Root transcriptome analysis of OsAS83 and OsAS84 revealed differential expression of many transcription factors involved in various signaling pathways, such as nutrient, pathogen, and stress responses, hormone signaling, development etc (Table 1). OsAS84 showed reduced expression of LOC_Os05g41540 (a bZIP transcription factor), LOC_Os05g14370.1 (WRKY82), LOC_Os02g42690 (a zinc finger, C3HC4 type domain containing protein) and LOC_Os06g33970.1 (a VQ domain containing protein) etc. Mutant of *Arabidopsis* homolog of LOC_Os05g41540, was significantly more sensitive to Zn depletion, and played role in uptake of Zn^67^. In plants, defense responsive pathways are regulated via several factors, and WRKY family proteins are one of the major regulators of this defense response^68^,^69^,^70^. Many of the WRKY genes are known to involved in jasmonic acid (JA) and salicyclic acid (SA) hormone mediated defense responsive pathways^71^. The expression of OsWRKY45, OsWRKY62, OsWRKY10, OsWRKY82, OsWRKY85, OsWRKY30, and OsWRKY83 was found to be responsive to SA and JA treatments^71^. Homolog of *OsWRKY82* in *Arabidopsis, WRKY70* was reported to be involved in osmotic stress, since double mutant of *wrky54 wrky70* exhibited enhanced tolerance to osmotic stress^72^. Overexpression of *ZmWRKY33* in *Arabidopsis* led to improved salt stress tolerance^73^. Overexpression of *Arabidopsis* homolog of LOC_Os02g42690, a E3 ubiquitin ligase *RING membrane-anchor 1* (Rma1), conferred drought tolerance in *Arabidopsis*^74^. *In silico* analysis of few of the selected genes showed differential expression in various anatomical tissues of rice. Among the analyzed genes, expression of LOC_Os10g39260 (*OsAPN*), LOC_Os02g10780 (*OsSPX2*), LOC_Os02g42690 (*OsZNC3HC4*), LOC_Os06g03770 (*OsABCT*) and LOC_Os05g41540 (*OsbZIP*) were found to have higher in root tissues, which further suggests their probable role in root development (Fig. S4 and Table S1). Differential expression of these genes in contrasting roots of OsAS84 and OsAS83 indicate their involvement in rice root development and physiology. As discussed above, these transcription factors may also be recruited by plants in various other signaling pathways like nutrient transport, hormone regulated pathogen response, stress response, and root development, as a more economic strategy.

### OsAS84 cultivar with advanced RSA shows less sensitivity to nutrient deficiency

**Figure 5:**
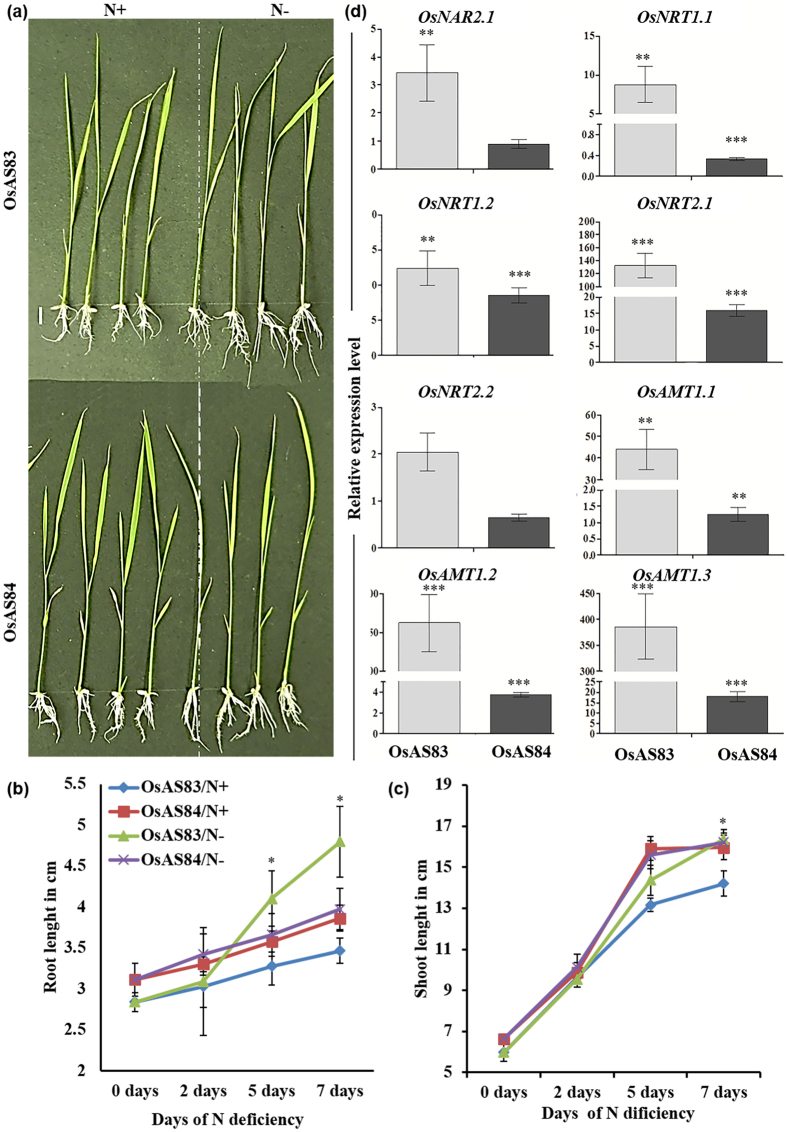
OsAS84 showed less sensitivity towards nitrogen deficiency. (**a**) Physiological responses of OsAS83 and OsAS84 to nitrogen deficiency. Scale bar 1 cm. (**b**,**c**) Primary root length and shoot length of OsAS83 and OsAS84 in N+ and N− conditions. Error bars represents standard error (n = 10). The experiment was repeated three times with similar results. (**d**) Expression pattern of *OsNRTs* and Os*AMTs* genes in root tissue harvested after 7th day, in both N deficit and normal conditions. Error bars indicate Standard error (n = 3). Asterisks indicate significant statistical differences, ***P < 0.001, **P < 0.01, *P < 0.05 (One-way ANOVA).

Increased RH density and stimulation of LR formation leads to enhanced nutrient uptake by increasing root surface area contact with soil. As OsAS84 RSA has higher number of CRs, high RH density in comparison to OsAS83, and differential expression of several transporters, we were interested to investigate the response of two varieties (with contrasting RSA) towards N and Pi nutrient deficit condition. Growth assay in N deficiency showed that RSA of OsAS84 was less sensitive in comparison to OsAS83, as indicated by increase in PR length by 2% and 38% increase, in case of OsAS84 and OsAS83, respectively (Fig. 5a and b). In case of shoot length, OsAS84 showed only 1.5% increase while OsAS83 showed 15% increase in comparison to nitrogen sufficient control condition (Fig. 5c).

Differential expression of nitrogen (N) and ammonium transporter affects the ability of root to absorb nitrogen. Ammonium and nitrate ions are the basic source of N for the plants, which are absorbed by ammonium transporter (AMT family) and nitrogen transports (NRT family). To further investigate the molecular reason for differential response of these two cultivars to N deficiency, the expression of some of the ammonium/nitrogen transporters and their protein partner *OsNAR2.1* was analysed. Rice seedlings of 3 dag were transferred to N sufficient and N deficit hydroponic media and RNA was isolated from the root tissues after 7^th^ day of treatment. The member of AMT family in rice, *OsAMT2.1,* encodes an ammonium transporter which is expressed in root^75^. *OsAMT1.1* and *OsAMT1.3* are involved in influx of NH4^+ ^76. *OsAMT1.1* and *OsAMT2.3* showed varied expression in roots at different doses of nitrogen^77^,^78^. In rice, *OsNRT2* (high-affinity) and a constitutively expressed *OsNRT1* (low affinity) were identified as nitrogen transporters^79^,^80^. Both AMTs and NRTs along with *OsNAR2.1* showed higher induction in expression level in OsAS83 in N deficiency (Figs 5d and S5). These results suggest that OsAS83 has to go through extensive transcriptional reprogramming to combat the N deficiency, which leads to drastic change in root growth pattern, while OsAS84 can withstand the N deficiency without any remarkable changes in root growth and transcriptional remodelling. This also suggests that root development and nitrogen transport mechanisms possess some common signalling crosstalk.

**Figure 6:**
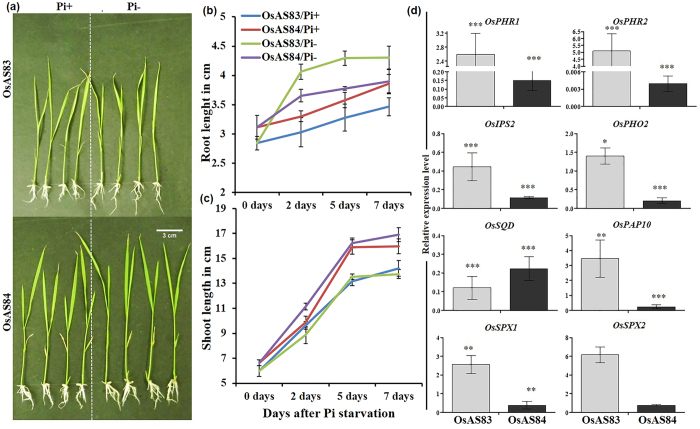
OsAS84 showed less sensitivity towards phosphate deficiency. (**a**) Physiological responses of OsAS83 and OsAS84 to Pi deficiency. Scale bar 1 cm. (**b**,**c**) Primary root and shoot length of OsAS83 and OsAS84 grown under Pi + and Pi- conditions. Error bars represent standard error (n = 10). The experiment was repeated three times with similar results. (**d**) Expression pattern of *PSI* genes in root tissue harvested after 7^th^ day, in both N deficit and normal conditions. Error bars indicates Standard error (n = 3). Asterisks indicate significant statistical differences, ***P < 0.001, **P < 0.01, *P < 0.05 (One-way ANOVA).

To enhance the capacity of plant to acquire more Pi from soil under Pi deficiency, the RSA gets altered showing formation of more LRs and increase in length and density of RH^81^,^82^. Growth assay under Pi deficiency showed that RSA of OsAS84 is less responsive in comparison to OsAS83, as PR length showed no difference and 24% increase, in case of OsAS84 and OsAS83, respectively (Fig. 6a and b). In OsAS84, presence of more root branches could be help to withstand the Pi starvation. In case of shoot length, OsAS84 showed only 5% increase while OsAS83 showed 4% decrease under Pi deficit condition (Fig. 6c). Plants adapt to low Pi by modulating the RSA for increasing topsoil foraging, through better Pi acquisition and efficient Pi utilization^83^. The adaptive strategies to cope up with Pi deficiency are tightly mediated by Pi signaling network and in rice, Pi starvation-induced signaling (PSI) is quite established. The central regulator of Pi signaling, *OsPHR1* and *OsPHR2* positively regulate *OsIPS1,* a non-protein coding gene which in turn sequesters *miR399*^84^. *miR399* post-transcriptionally negatively regulates *OsPHO2*, which is involved in the enhancement of uptake and translocation of Pi. In root, *OsSPX1* also negatively regulates *OsIPS1* and other members of SPX family under Pi-supplied conditions^85^.

Since OsAS83 and OsAS84 cultivars showed significantly variability in terms of alteration of RSA in response to Pi deficiency, we also investigated the expression of PSI genes in roots of both the cultivars. Rice seedlings at 3 dag were transferred to Pi sufficient and Pi deficit hydroponic media and RNA was isolated from the root tissues after 7^th^ day of treatment. *OsSPX2* expression was highly induced in Pi sufficient conditions in OsAS84 roots, while in Pi deficiency, expression of *OsSPX2* was found to be more induced in OsAS83 roots, which is also true in case of other PSI genes such as *OsPHO2, OsPHR2, OsPAP10,* and *OsSQD* (Fig. 6d and Fig. S6). This indicates that both varieties have differential regulation of PSI signaling pathway. *OsSPX1* negatively regulates Pi accumulation in shoots and acts as a positive regulator of *OsSPX2, OsSPX3* and *OsSPX5*^85^. The expression of *OsSPX1* was lower in Pi sufficient conditions and the level of Pi in shoot was higher in case of OsAS84 (Fig. S6), which indicates that *OsSPX1* negatively regulates Pi accumulation in OsAS84 similar to previous published report^85^. Level of induction of *OsSPX1* was also lower in OsAS84 roots, in comparison to OsAS83, under Pi starvation. The central regulator of PSI signaling, *OsPHR1* and *OsPHR2,* showed higher level of expression in roots of OsAS84 under Pi sufficient conditions, however, under Pi starvation, the expression level of these two genes was downregulated (Fig. 6d). The level of *OsIPS2* expression was more in OsAS84 roots under Pi sufficient condition while its expression level was reduced more in OsAS84 under Pi deficit conditions (Fig. 6d and Fig. S6). Target of rice *miR399, OsPHO2* also followed a similar pattern of expression (Fig. 6d). These results suggest that OsAS84 is more resistant to Pi starvation, which is possibly due to naturally existing more robust RSA than OsAS83. This also suggests that plants recruit various common regulators for RSA trait and PSI pathway and that nutrient acquisition and RSA trait are strongly linked at molecular level.

### OsAS84 with advanced RSA shows increased resistance to abiotic stresses

**Figure 7:**
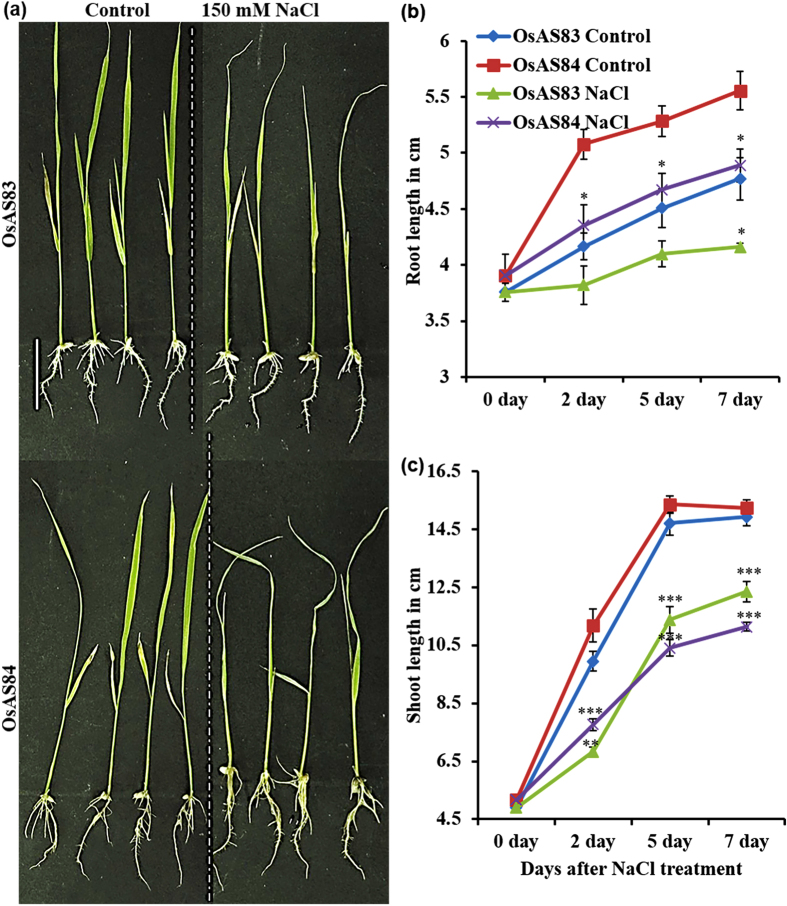
OsAS83 and OsAS4 shows variable physiological responses under salinity stress. (**a**) Phenotypic variation in both the cultivars after treatment of 150 mM NaCl for 7 days. Scale bar 1 cm. (**b**,**c**) Primary root and shoot length of OsAS83 and OsAS84 at 0 days, 2 days, 5 days and 7 days after treatment. Error bars represents standard error (n = 10). The experiment was repeated three times with reproducible results. Asterisks indicate significant statistical differences, ***P < 0.001, **P < 0.01, *P < 0.05 (One-way ANOVA).

Several genes related to dehydration, salt or oxidative stress tolerance were found to be upregulated in OsAS84 roots, for *e.g*. genes involved in trehalose and ethylene biosynthesis, peroxidase, aspartic proteinase, ERE-binding protein, and NADH dehydrogenase. Abundant expression of these genes in advanced RSA of OsAS84 are indicative of its possible resistance to abiotic stresses. To test this hypothesis, we first analyzed the response of these two cultivars toward salinity stress. When 3 dag old seedlings of both cultivars were subjected to high salt conditions, OsAS84 showed less sensitivity to salinity stress, as evident by less wilting and yellowing in leaves in comparison to OsAS83 (Fig. 7a). Overall root growth was also found to be less affected in OsAS84. Development of seminal/CRs was less affected in OsAS84 than OsAS83 (Fig. S7). The PR length was reduced by 13% and 12%, while shoot length was reduced by 18% and 27% in OsAS83 and OsAS84, respectively (Fig. 7b and c). Under salinity stress, despite decrease in PR length, OsAS84 showed healthy shoots, which could be due to increased number and length of CR and LR. In OsAS84, the shoot length was reduced as a primary response to the salinity stress (osmotic phase), but the leaves showed less wilting and yellowing, and rate of dying was also slower (ion-specific, phase) in comparison to OsAS83^86^. These observations suggest that OsAS84 cultivar having robust RSA is more resistance to salinity stress than OsAS83, and indicate a positive correlation between RSA and stress response.

**Figure 8:**
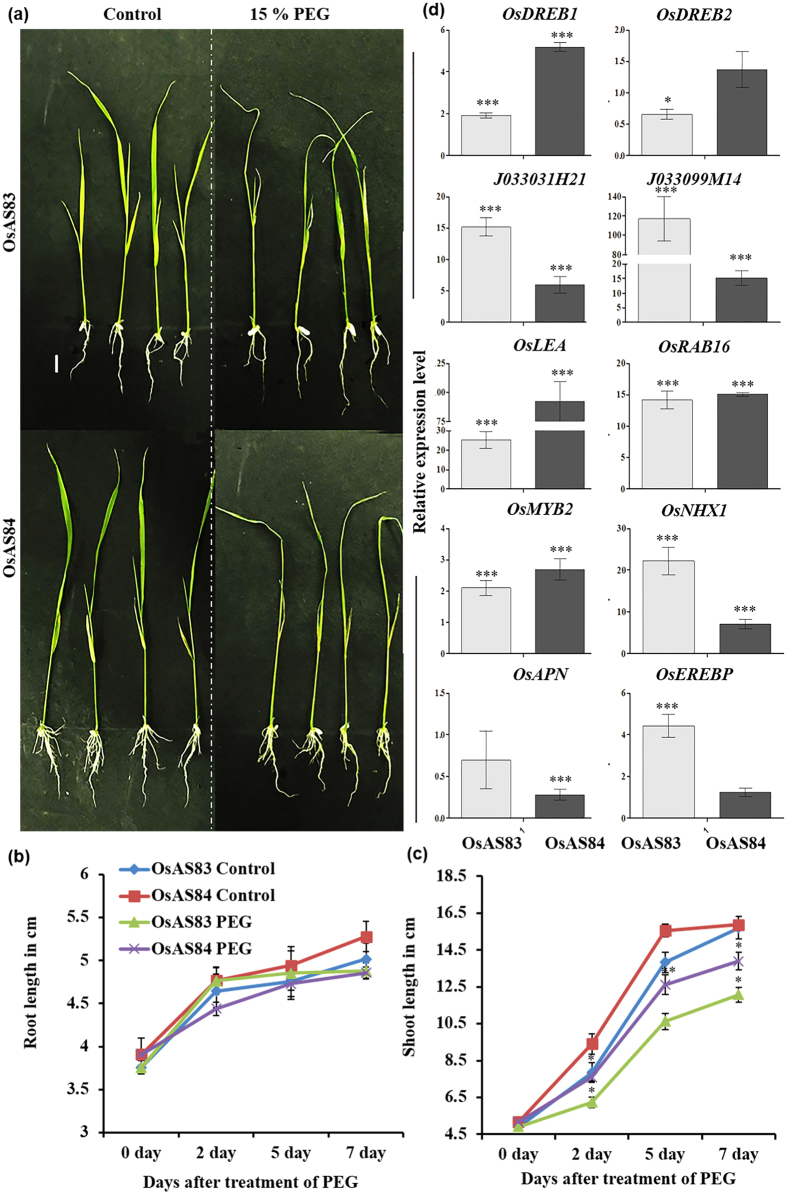
OsAS84 showed higher resistance to dehydration stress than OsAS83. Physiological responses of OsAS83 and OsAS84 upon dehydration stress. (**a**) 3 dag seedlings of both the cultivars were treated with 15% Polyethylene glycol 6000 (PEG 6000) for 7 days in hydroponics. Scale bar 1 cm. (**b**,**c**) Primary root and shoot length of OsAS83 and OsAS84 in control and PEG conditions at 0, 2, 5, and 7 days. Error bars represents standard error (n = 10). The experiment was repeated three times with reproducible results. (**d**) Expression of dehydration responsive genes in root tissue harvested after 7 day of treatment. Error bars represent Standard error (n = 3). Asterisk indicates significant statistical differences, ***P < 0.001, **P < 0.01, *P < 0.05 (One-way ANOVA).

Then, we analyzed the response of these two cultivars to water deficit conditions. When 3 days old seedlings were subjected to dehydration stress (Fig. 8a and Fig. S8a), we observed 8% and 4% reduction in PR length and 12% and 24% reduction in shoot length in case of OsAS84 and OsAS83, respectively (Fig. 8b and c). Besides decrease in PR length, overall RSA of OsAS84 was found to be healthy (more branches) than OsAS83 under dehydration stress. Leaves of OsAS84 were found to be less affected by dehydration stress in comparison to OsAS83. These results indicate that OsAS84 with advance RSA is comparatively less sensitive to dehydration stress than OsAS83. We further analyzed if this dehydration response accompanied changes in the expression of relevant genes in roots of these cultivars. For this, 3 dag seedlings of OsAS83 and OsAS84 were transferred to control and 15% PEG containing hydroponic media and qRT-PCR was done using cDNA prepared from RNA isolated from the root tissues after 7^th^ day of treatment. We observed that some of the dehydration responsive genes like *OsMYB2, LATE EMBRYOGENESIS ABUNDANT*(*OsLEA*), *DEHYDRATION-RESPONSIVE ELEMENT (OsDREB1*) and *OsDREB2* were upregulated in OsAS84 roots in comparison to OsAS83, while *OsNHX1* (maintain homeostasis in salt and water stress) and amino acid kinase genes, *J033099M14* and *J033031H21* (proline biosynthesis genes) were downregulated in OsAS84 under dehydration stress (Fig. 8d). Plants used several mechanisms to combat dehydration stress, such as ABA dependent/independent and DREB mediated signaling and accumulation of osmotolerant compounds like proline or glycine betaine. Upregulation of *OsMYB2, OsLEA, OsDREB1* and *OsDREB2* genes suggests that upon exposure to dehydration stress OsAS84 activates *OsDREB2-* dependent signaling while genes involved in proline accumulation were not induced in OsAS84 upon dehydration stress, indicating differential signaling pathways respond differently in OsAS83 and OsAS84 upon dehydration stress.

Under normal condition, dehydration responsive genes were upregulated in OsAS84 in comparison to OsAS83 (Fig. S8b). In our microarray analysis, dehydration responsive genes such as LOC_Os10g39260 (*OsAPN)* and LOC_Os02g43840 (*OsEREBP)* were differentially expressed in OsAS84 roots. Expression of both of these genes was higher in OsAS84 in normal conditions, but their expression was higher in OsAS83 (Fig. 8d and Fig. S8b) under dehydration stress. This indicates that *OsAPN* and *OsEREBP,* along with other dehydration responsive genes, probably act as molecular regulators for enhanced response towards dehydration stress in OsAS84. The expression pattern of dehydration responsive genes, under control and dehydration stress, indicate these genes play important role in conferring resistance to dehydration stress, and that sensitive OsAS83 with poor RSA had to undergo more rigorous changes at the relevant transcript level to combat dehydration stress through changes in RSA and physiology.

### Phytohormones differentially regulates root growth in two cultivars

It is well known that phytohormones play very critical role in development of PR as well as CRs/LRs in rice or *Arabidopsis*. It has been shown previously with *avr1* and *crl1* mutants studies in rice that auxin promotes initiation of CRs^13^. Cytokinins, which act antagonistically to auxin, also play important role in controlling the initiation of LR primordia by affecting auxin distribution^87^,^88^,^89^. Cytokinin signaling works through *WOX11* and *ARABIDOPSIS RESPONSE REGULATORS) (ARRs*) in rice and overexpression of *OsRR6* (CK-inducible type-A response regulator) led to decreased LR outgrowth^90^. Abscisic acid (ABA) is known to regulate LR development differently under normal (low level of exogenous ABA) and stressed conditions^91^. Ethylene has also been reported to influence the LR development by stimulation of auxin biosynthesis^92^,^93^.

To understand the effect of phytohormones on RSA of two contrasting rice cultivars, we analyzed the difference in root and shoot growth pattern between two cultivars after treatment with various phytohormones (1 μM ABA, BAP, IAA and GA (Fig. 9S). Treatment of 1 μM ABA led to enhancement of root and shoot growth in OsAS83 by 33% and 7%, respectively. With same treatment in OsAS84, we observed 3% increase and 20% decrease in root and shoot length, respectively (Fig. S9b). ABA treatment also increased LR number and length in OsAS83 in comparison to control plant. In BAP (a CK) treatment, both the cultivars showed reduction in root and shoot growth, but OsAS84 show more reduction in both root (14%) and shoot growth (7%) in comparison to OsAS83, in which only 2.5% reduction was observed in root growth after 7 days of treatment (Fig. S9c). IAA showed stimulatory effect on root growth in both cultivars (2% increase in root length), since low dose of auxin is known to promote root growth (Fig. S9d). GA led to significant increase in shoot length by 118% and 96% in OsAS83 and OsAS84, respectively. However, treatment with 1 μM of GA led to 10% increase in root growth in OsAS83 and 18.5% reduction in root growth in OsAS84 (Fig. S9e). Although a matter of further study, the differential expression of several hormonal regulatory pathways related genes (such as, LOC_Os02g43840, LOC_Os11g13670, and LOC_Os06g07040) in roots of two cultivars (with contrasting RSA) may contribute to their variable responses to phytohormones. Similarly, hormonal regulatory pathway genes may also contribute largely to the variation in RSA trait observed among rice cultivars.

In the present study we have identified and analyzed two *indica* rice cultivars - OsAS83 and OsAS84, which differ significantly in their RSA traits and responses towards abiotic stresses and nutrient deficiency. Based on our gene expression microarray studies and marker analysis, we propose that the stable contrasting variation in RSA trait between these two cultivars is potentially conferred by the differential expression of various transcripts in their root. Both the cultivars (with robust or shallow RSA) behaved differently in nutrient deficiency and in abiotic stresses. We propose that longer PR, more number of CRs/LRs, and higher RH density makes OsAS84 cultivar more adaptive to nutrient deficiencies and other abiotic stresses (salinity/dehydration). Differential expression pattern of nitrogen transporters, PSI signaling genes and dehydration responsive genes in roots of rice cultivars indicates a strong correlation between molecular regulation of RSA trait and physiological responses in rice. Our results also indicate that plants may recruit its common molecular machinery, at least in part, for different regulatory pathways like root development, nutrient signaling, abiotic stresses or pathogen responses etc. Further elaborate studies of this common regulatory mechanism could be helpful to develop rice varieties with improved developmental and physiological traits of agronomic importance.

## Methods

### Plant materials, growth conditions and treatment

For rice study, IR-64 and IET-16348 seeds were germinated and grown in hydroponics media under controlled conditions (16 h:8 h::light:dark cycle, 28 °C ± 1 °C, light intensity 250 μmol m^−2^s^−1^)^94^. For Pi and N deficiency, seedlings were kept in hydroponics Yoshida medium lacking K_2_HPO_4_/KH_2_PO_4_ stocks and NH_4_NO_3_ stock, respectively^94^. For dehydration and salinity stress, hydroponics medium was supplemented with 15% of PEG 6000 and 150 mM NaCl, respectively. For hormone treatment, rice seedlings were grown in hydroponics media supplemented with various hormones (1 or 10 μM of ABA, BAP, GA, and IAA).

### Detection of macro or micro elements

Plants were raised in laterite soil with farmyard manure (6:1 ratio) having a water holding capacity of 22%. Recommended dose of fertilizer was added to the soil in the form of urea, super phosphate and murate of Potash. Plants were harvested on 35^th^ day for analysis. Samples were ground to a fine powder and acid digested using nitric acid and perchloric acid. Double distilled water was added to the digested samples and filtered using 0.45 μm filter paper and a vacuum pump. These samples were analyzed for metals using ICP-OES (Thermo Scientific iCAP 6000). In this, liquid samples are injected and atomic emission was recorded. Measurements were made using monochromator/photomultiplier combination for P and polychromator and an array detector combination for the Mg, K, Fe, Zn, and S.

### RNA isolation and qRT-PCR

RNA was isolated from the different tissues of rice seedling using the TRI-reagent (Sigma) at different time intervals and was further treated with RNase-free DNase I (Thermo Scientific) as per manufacturer’s instructions. cDNA preparation and qRT-PCR was performed using the previously described method^95^. After checking the quality and spectrophotometric quantification, 2 μg RNA were converted into cDNA using M-MLV Reverse Transcriptase (Invitrogen, USA) following the manufacture’s protocol. Each sample was analyzed in triplicate using at least two cDNA preparations by qRT-PCR using 2X Brilliant II SYBR® Master Mix (Agilent Technologies, USA). PCR reaction was placed in ABI PRISM 7900 HT Fast Real Time PCR System (Applied Biosystems, USA). The specificity of the reactions was verified by carrying out melting (dissociation) curve analysis. *GLYCERALDEHYDE-3-PHOSPHATE DEHYDROGENASE (GAPDH*) and *ACTIN 11* were used as endogenous control. Primer sequences, which were used in study, are given in Table S2.

### Gene chip Microarray

Total RNA was extracted from 14 days old root tissue of OsAS83 and OsAS84. Quality of total RNA was analyzed by Agilent 2100 Bioanalyser and RIN values were above 7. RNA (250 ng) was used from each sample in two biological replicates for setting up *in-vitro* transcription reaction; RNA purification, fragmentation and hybridization reaction was done using *O. sativa* gene chip arrays (Affymetrix). The array contains 51,279 independent probes corresponding two rice cultivars *japonica* and *indica* (http://www.affymetrix.com). Washing and scanning were followed as suggested in Affymetrix Gene Chip total RNA procedure. The processed raw signal intensities were subjected to normalization using the Gene spring GX software v11.5. Result obtained after scanning were analyzed using Gene Spring software. Expression of selected genes differentially expressed in microarray was further validated through real time qRT-PCR (as mentioned above).

### Histological analysis

Primary root tips (0–1 cm) of 2 days old seedlings of OsAS83 and OsAS84 were serially sectioned by free hand. Sections were stained with 0.1% Safranin stain (Sigma) and were visualized under bright field microscope (Nikon 80i). Cell size and diameter was calculated using I_MAGE_J software.

## Additional Information

**How to cite this article**: Singh, A. *et al*. Root transcriptome of two contrasting *indica* rice cultivars uncovers regulators of root development and physiological responses. *Sci. Rep.* **6**, 39266; doi: 10.1038/srep39266 (2016).

**Publisher's note:** Springer Nature remains neutral with regard to jurisdictional claims in published maps and institutional affiliations.

## Supplementary Material

Supplementary Figures and Tables

## Figures and Tables

**Table 1:** Genes differentially expressed in OsAS84 in comparison to OsAS83 roots.

Accession No.	Fold change in microarray	*Arabidopsis* ID	Function	References
**Transporters**				
LOC_Os03g29850.1	2.7012017	AT1G55910	Metal cation transporter	39
LOC_Os05g14820.1	2.8707578	AT5G40780	Transmembrane amino acid transporter protein	96
LOC_Os09g20490.1	2.8190773	AT1G78130	Transporter, putative	
LOC_Os03g40780.1	2.158206	AT1G49870	Transport protein-related, putative	
LOC_Os02g02170.1	16.11485	AT1G08090	Transporter, major facilitator family, putative	
LOC_Os12g29950.1	2.418649	AT2G39210	Nitrate chloride transporter	
LOC_Os07g31884.1	2.2769732	AT3G23560	MATE efflux family protein, putative	97
LOC_Os05g12320.1	−2.959296	AT5G53190	Nodulin MtN3 family protein, putative	53
LOC_Os07g03960.1	−3.7330253	AT5G61520	Transporter family protein, putative	
LOC_Os12g08090.1	−4.244538	AT1G77380	Amino acid transporter, putative	98
LOC_Os06g03770.1	−2.0081406	AT5G58270	ABC transporter, ATP-binding protein, putative	99
LOC_Os08g41590.1	−6.2401733	AT1G72125	Peptide transporter PTR2, putative, expressed	100
LOC_Os01g17214.1	−2.0424147	AT3G43790	Major facilitator superfamily antiporter, putative, expressed	101
**Hormone signaling related**				
LOC_Os09g27820.1	19.6028	AT1G05010	1-aminocyclopropane-1-carboxylate oxidase protein, putative, expressed	102
LOC_Os02g43840.1	4.617959	AT5G47310	Ethylene-responsive element-binding protein, putative, expressed	103
LOC_Os11g13670.1	6.153131	AT5G23530	Gibberellin receptor GID1L2, putative, expressed	
LOC_Os07g40240.1	−5.867474	AT5G59845	GASR9 - Gibberellin-regulated GASA/GAST/Snakin family protein precursor, expressed	60
LOC_Os06g07040.1	−3.1850903	AT2G33310	OsIAA20 - Auxin-responsive Aux/IAA gene family member, expressed	104
LOC_Os11g29120.1	−15.173324	AT1G04910	Growth regulator related protein, putative, expressed	
LOC_Os05g41760.1	−2.0487518	AT1G28360	AP2 domain containing protein, expressed	105
**Transcription factors**				
LOC_Os05g41540.1	−2.7472656	AT2G16770	bZIP transcription factor domain containing protein, expressed	67
LOC_Os05g14370.1	−5.4798274	AT3G56400	WRKY82, expressed	106
LOC_Os06g33970.1	−2.890836		VQ domain containing protein	91
LOC_Os02g42690.3	3.7169807	AT4G03510	Zinc finger, C3HC4 type domain containing protein,	107
LOC_Os03g24970.1	3.0067194	N/A	SWIM zinc finger family protein	
LOC_Os07g29770.1	2.1135173	AT1G70150	Zinc finger protein, putative, expressed, ATROPGEF7/ROPGEF7	
LOC_Os06g07020.1	−2.8766186	AT5G52010	ZOS6-01 - C2H2 zinc finger protein, expressed	
LOC_Os11g47630.1	−2.201833	AT3G10470	ZOS11-10 - C2H2 zinc finger protein, expressed	
