# Supplementary material for: Root transcriptome of two contrasting indica rice cultivars uncovers regulators of root development and physiological responses
Source: Sci Rep. 2016 Dec 21;6:39266. doi: 10.1038/srep39266 (PMC5175279; doi:10.1038/srep39266)
Supplement: Supplementary Figures and Tables [file srep39266-s1.pdf]

Title:

Root transcriptome of two contrasting *indica* rice cultivars uncovers regulators of root development and physiological responses

Running title:

Molecular regulation of rice root architecture

Authors:

Alka Singh[$1], Pramod Kumar[$1], Vibhav Gautam[1], Balakrishnan Rengasamy[1], Bijan Adhikari[2], Makarla Udayakumar[3] and Ananda K. Sarkar*[1]

[$]These authors have contributed equally.

Affiliation:

[1]National Institute of Plant Genome Research, Aruna Asaf Ali Marg, New Delhi 110067, India.

[2]Dr. Bijan Adhikari, Rice Research Station, Chinsurah, West Bengal 712101, India.

[3]Department of Crop Physiology, University of Agricultural Sciences, GKVK, Bangalore 560065, India

Supplemental figures

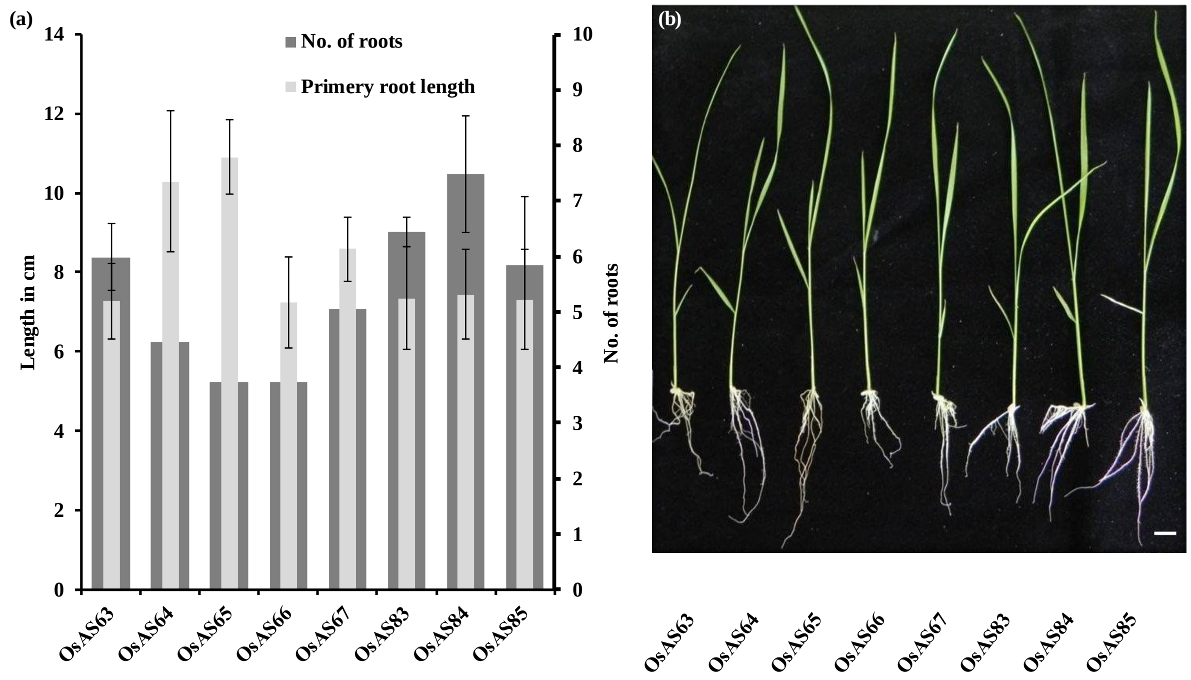

Supplemental figure S1. **Growth assay of various rice cultivars.**
(a-b) Growth assay of various rice cultivars in hydroponics medium at 14 dag (n = 10). Scale bar 1 cm.

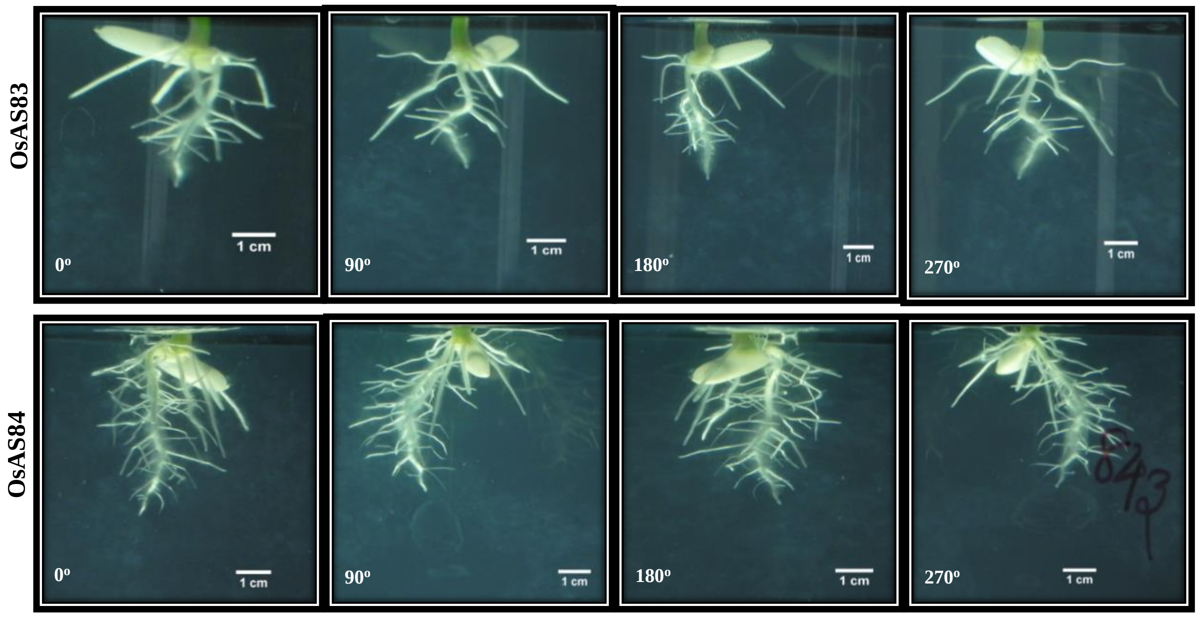

Supplemental figure S2. **Visualization of OsAS84 and OsAS83 at differ angles.**

To visualize the RSA, plants were grown in hydroponic medium for 7 days. Pictures were captured at different angle. Scale bar 1 cm. OsAS84 shows high root density than OsAS83.

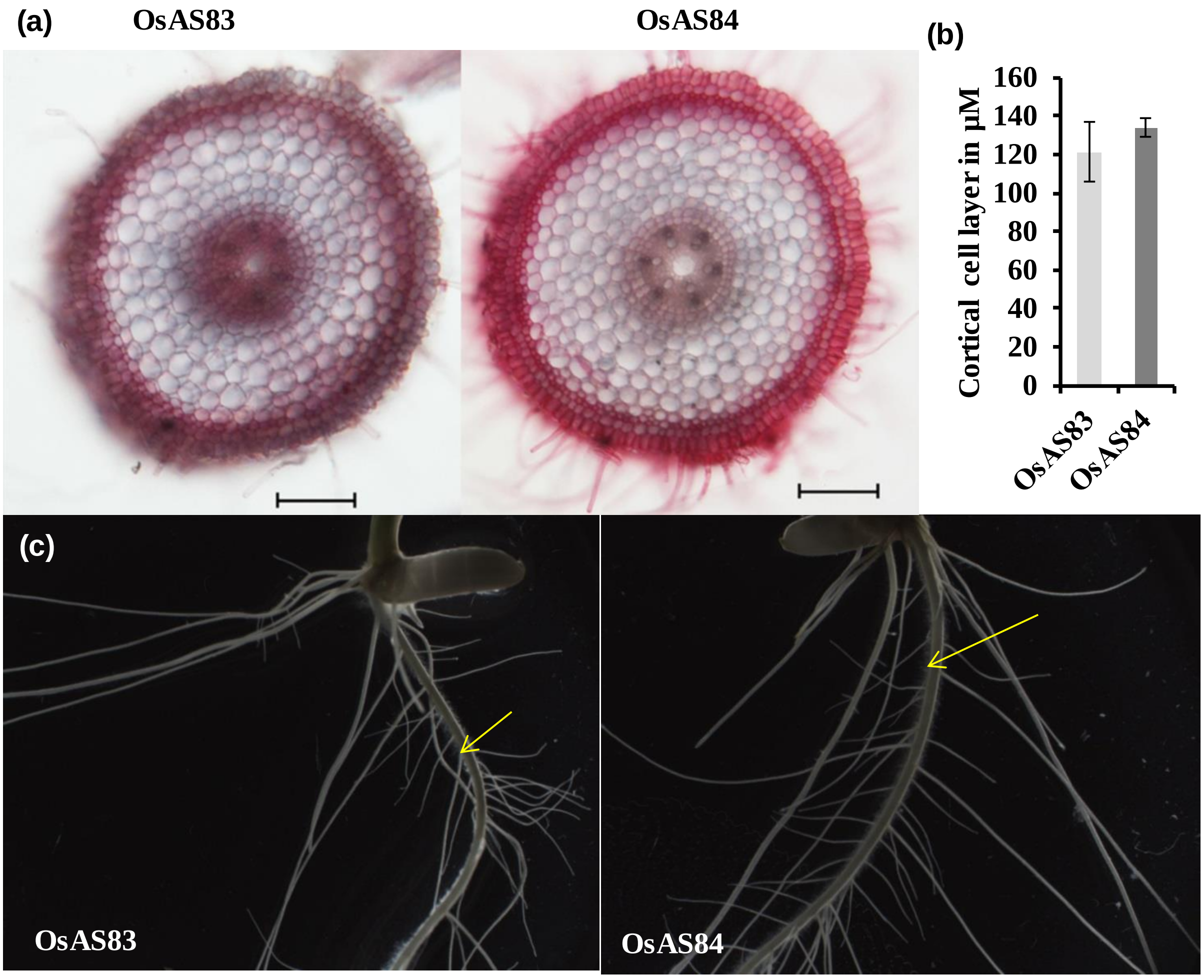

Supplemental figure S3. OsAS84 and OsAS83 differ in their histological characters.
(a) Transverse sections of 2 days old root from 1 cm of root tip. Images shown are representative of at least 10 sections. Scale bar 100 µm. (b) Measurement of cortical layer in OsAS83 and OsAS84. Error bar represent standard error (n = 10). (c) Enlarge view of OsAS83 and OsAS84 root of 5 dag. Arrow indicates root hair density.

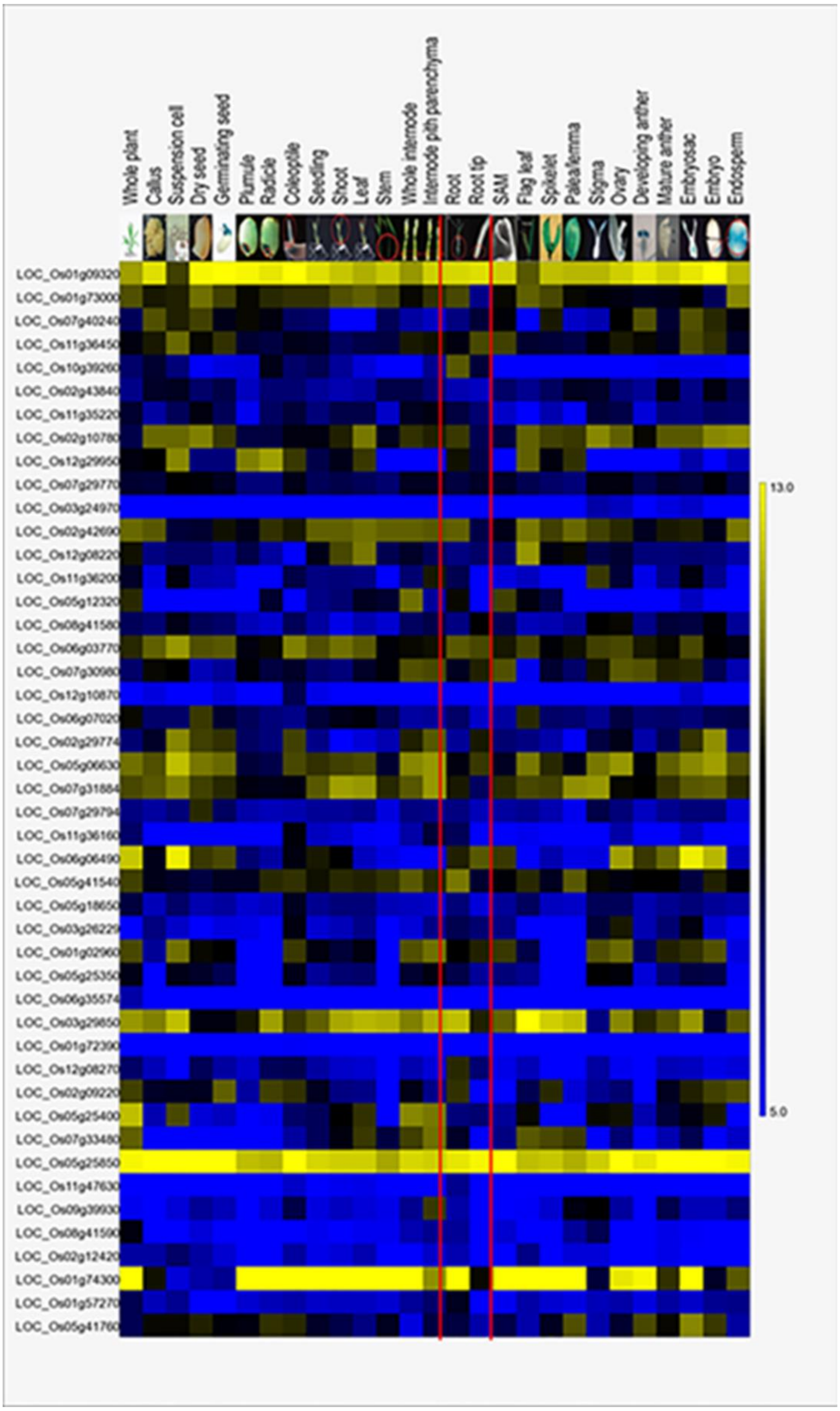

Supplemental figure S4. ***In silico* expression profiling of selected genes in different anatomical stages of rice.**

Validated genes were analyzed *in silico* for their tissue specific expression by Rice Oligonucleotide Array DataBase (http://www.ricearray.org).

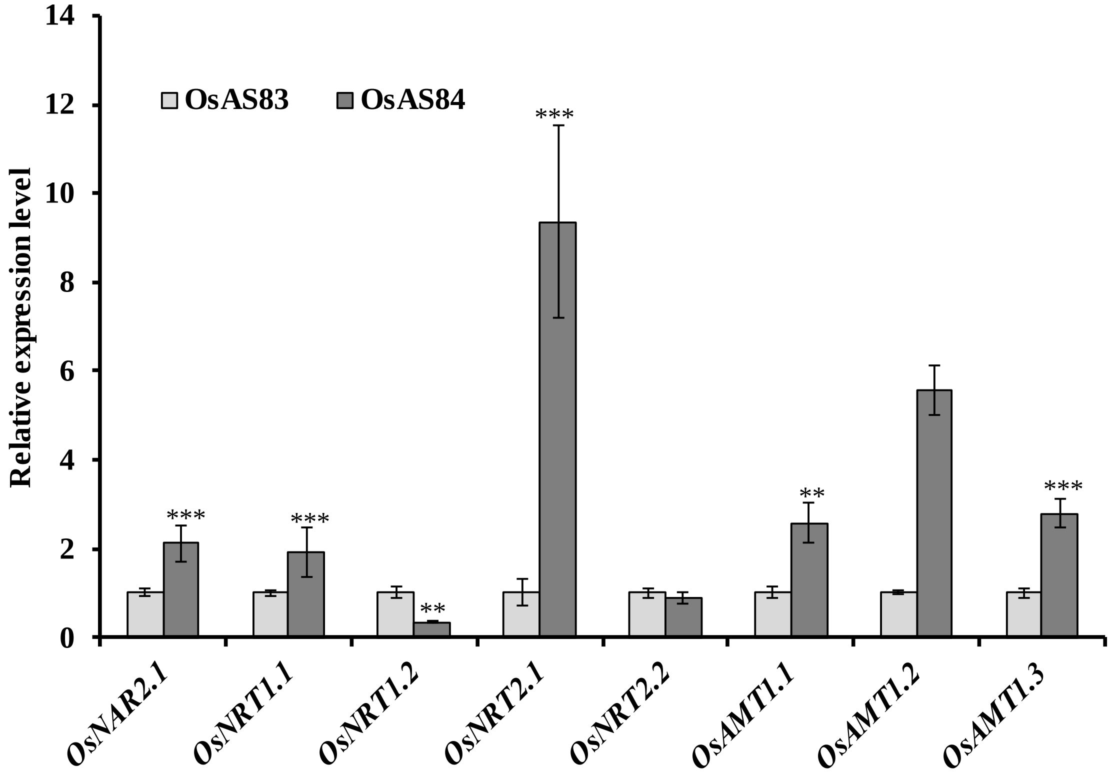

Supplemental figure S5. Nitrate and ammonium transporters showed increased expression in OsAS84 in N sufficient (normal) condition.

Expression analysis of nitrogen and ammonium transporters in root of OsAS83 and OsAS84. Rice seedlings were germinated for 3 days on wet paper and transferred to hydroponics medium. Root samples were harvested after 7 days. Mean of three independent biological replicate was plotted. Error bars indicate SE (n = 3). Asterisks indicate significant statistical differences, ***P< 0.001, **P< 0.01, *P< 0.05 (One-way Anova).

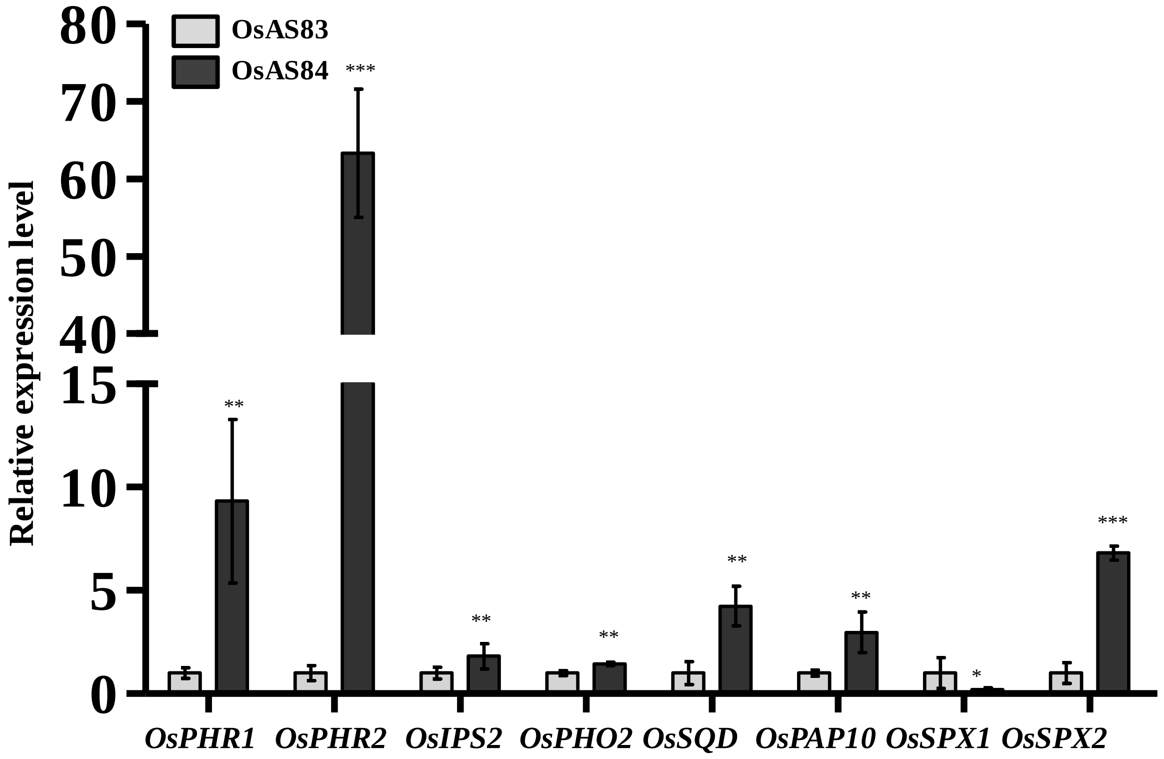

Supplemental figure S6. **Phosphate starvation- inducible genes (PSI) showed increased expression in OsAS84 in Pi sufficient (normal) condition.**
Expression analysis of PSI genes in root of OsAS83 and OsAS84. Rice seedlings were germinated for 3 days on wet paper and transferred to hydroponics medium. Root samples were harvested after 7 days. Mean of three independent biological replicate was plotted. Error bars indicate SE (n = 3). Asterisks indicate significant statistical differences, ***P< 0.001, **P< 0.01, *P< 0.05 (One-way Anova).

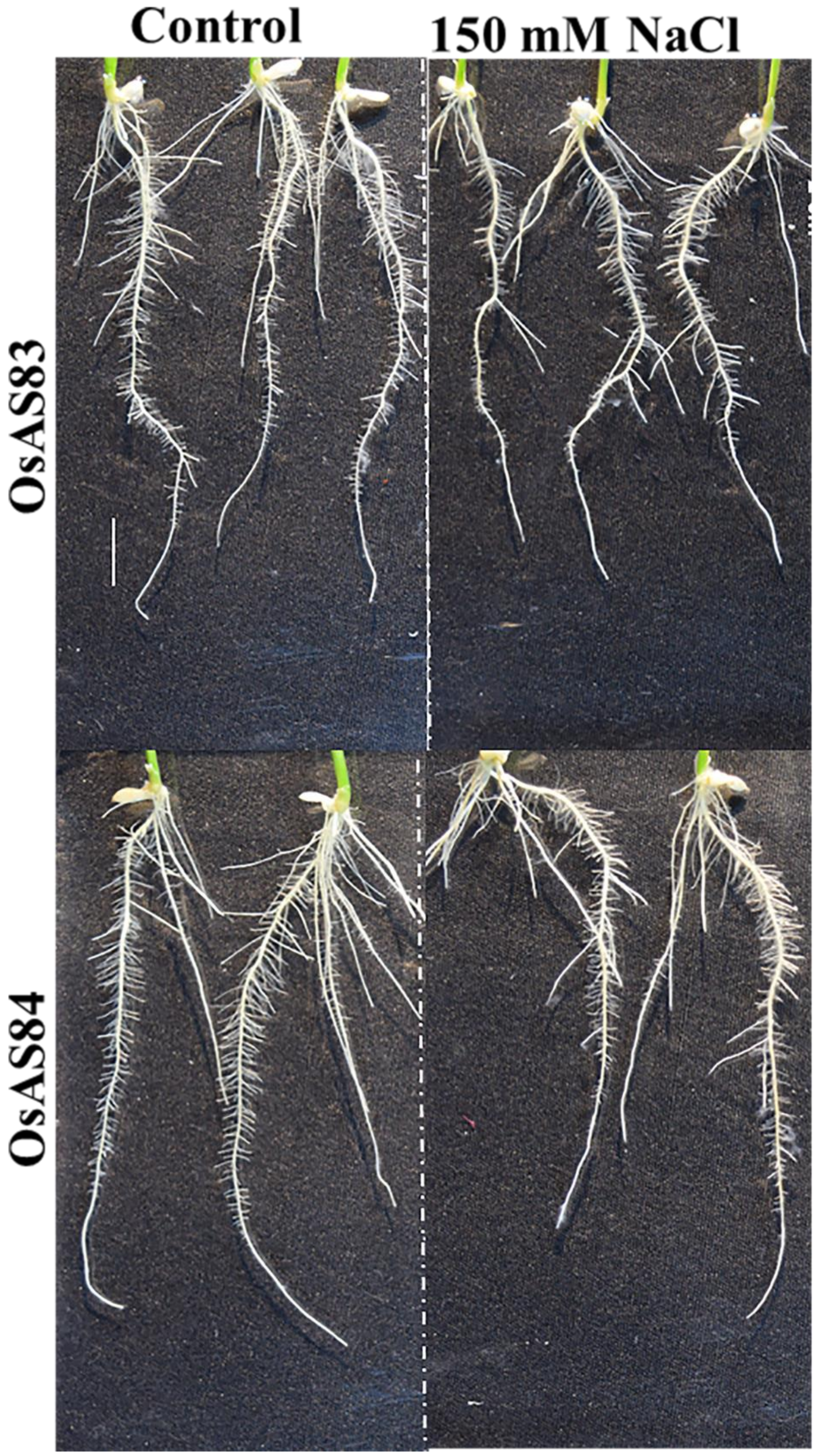

Supplemental figure S7. OsAS83 and OsAS84 showed variation in root growth on salinity stress.
Enlarge view of root of OsAS83 and OsAS84 after treatment of NaCl.
Rice seedlings were germinated for 3 days on wet paper and transferred to hydroponics medium containing 150 mM NaCl. Root samples were harvested after 7 days. Scale bar 1 cm.

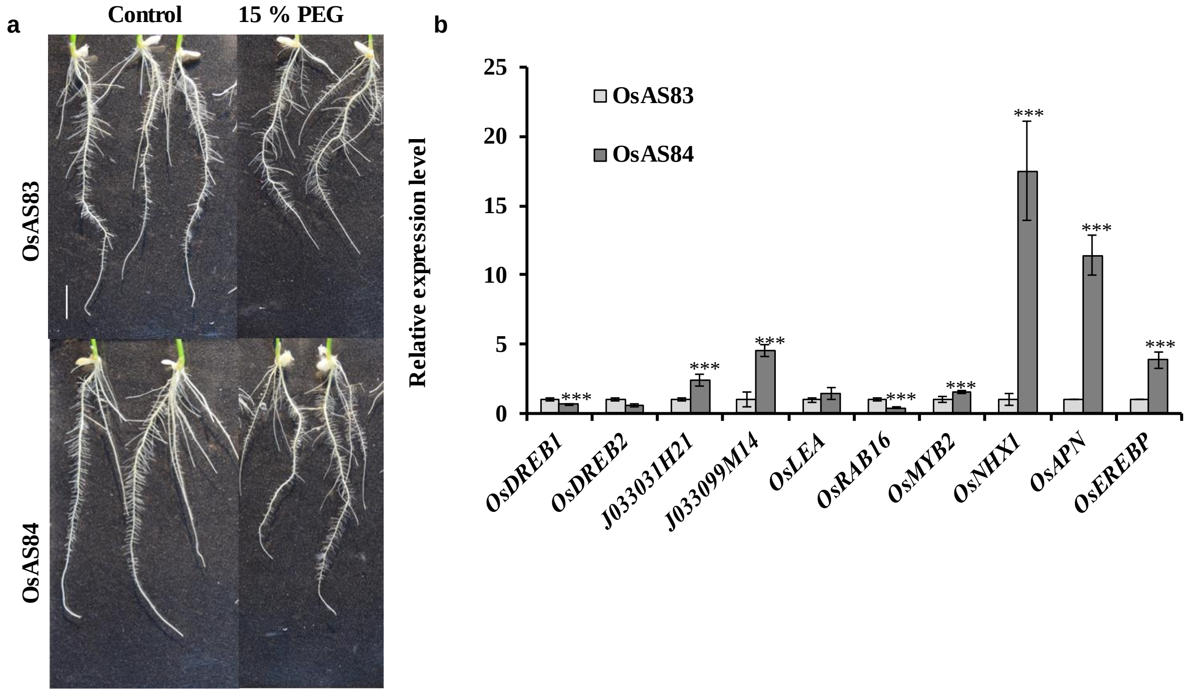

Supplemental figure S8. OsAS83 and OsAS4 showed differential phenotypic variation in root morphology under dehydration stress.

(a) Enlarge view of root of OsAS83 and OsAS84 after treatment of PEG. Rice seedlings were germinated for 3 days on wet paper and transferred to hydroponics medium containing 15% PEG 6000. Root samples were harvested after 7 days. Scale bar 1 cm. (b) Expression analysis of dehydration responsive genes in normal conditions. Mean of three independent biological replicate was plotted. Error bars indicate SE (n = 3). Asterisks indicate significant statistical differences, ***P< 0.001, **P< 0.01, *P< 0.05 (One-way Anova).

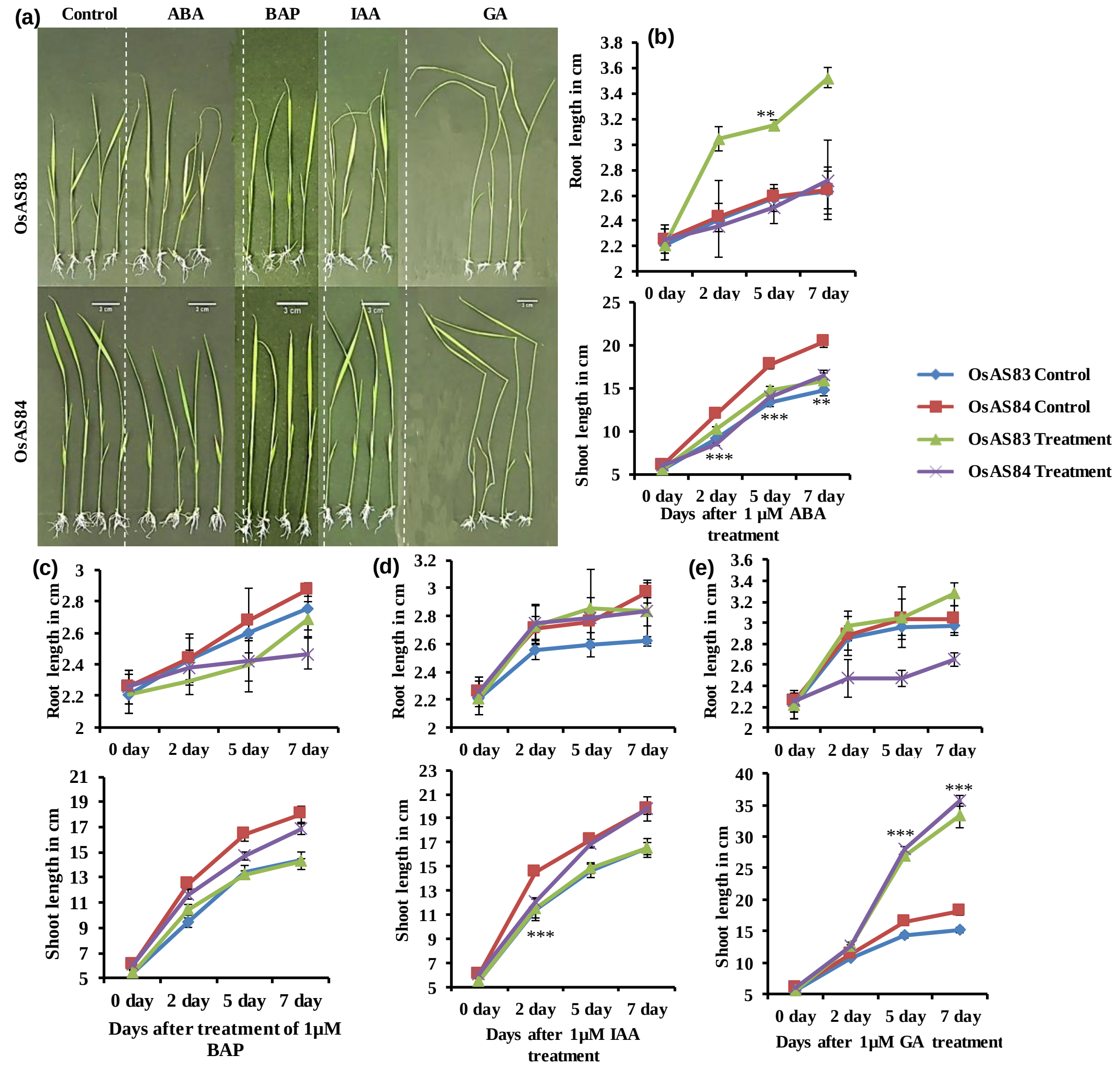

Supplemental figure S9. Phytohormones treatment leads to altered root and shoot morphology.

Rice seedlings were germinated for 3 days on wet paper and transferred to hydroponics medium containing 1 µM ABA, 1 µM BAP, 1 µM IAA and 1 µM GA. Root samples were harvested at 0, 2, 5 and 7 days. (a) Phenotype of OsAS83 and OsAS84 seedlings after treatment with phytohormones. Scale bar 1 cm. (b) Primary root length and shoot length after treatment of 1 µM ABA. (c) Primary root length and shoot length after treatment of 1 µM BAP. (d) Primary root length and shoot length after treatment of 1 µM IAA. (e) Primary root length and shoot length after treatment of 1 µM GA. In b-e, error bars represent standard error (n=10). Experiment repeated 3 times with similar results. Asterisks indicate significant statistical differences, ***P<0.001, **P<0.01, *P<0.05 (One-way Anova).

Table S1. List of validated genes by real time qRT-PCR.

Gene Id	FC in Real time qRT-PCR	FC in microarray	Putative function
LOC_Os07g29794	110.5276525	2.8709397	Calmodulin binding heat shock protein, putative,
LOC_Os02g09220.1	69.76763704	8.314912	Cytochrome P450
LOC_Os05g18650	27.83658991	9.414617	Expressed protein
LOC_Os06g35574.1	25.84187264	2.4708936	mki67 protein, putative, expressed
LOC_Os06g30920	8.14923581	5.000838	
LOC_Os03g24970	5.731105462	3.0067194	SWIM zinc finger family protein
LOC_Os02g43840	3.244021143	4.617959	EREBP-4 like protein
LOC_Os11g35220	3.23464264	5.0498934	OsWAK117 - OsWAK receptor-like cytoplasmic kinase
LOC_Os05g06630	3.118850356	16.080547	Serine peptidase, protein binding
LOC_Os12g08270.1	3.066393829	3.2962341	Inositol-1-monophosphatase
LOC_Os01g73000	2.318126223	53.4	Response to oxidative stress, peroxidase
LOC_Os12g29950	1.904615928	2.4	Nitrate Chloride transporter
LOC_Os03g29850	1.884653866	2.7012017	Metal Cation transporter
LOC_Os10g39260	1.757361508	5.4163294	Aspartic proteinase nepenthesin
LOC_Os07g31884	1.718261814	2.2769732	MATE efflux ALF5
LOC_Os01g72390.1	1.589937991	6.5760393	NBS type disease resistance protein
LOC_Os02g10780	1.398431577	6.11331	SPX domain containing protein
LOC_Os01g09320	1.271756759	15.6	NADP-dependent malic enzyme
LOC_Os02g29774	1.170207741	12.615214	N/A
LOC_Os02g42690	1.141915757	3.7169807	Zinc finger, C3HC4 type domain containing protein
LOC_Os12g08220	1.138394117	2.1608307	Arabidopsis Thaliana Histone Deacetylase 14 (Hda14)
LOC_Os07g29770	1.100331237	2.1135173	Zinc finger protein, putative
LOC_Os09g39930.1	-1.027619995	-2.620259	Tyrosine protein kinase
LOC_Os05g41540	-1.06650036	-2.7472656	bZIP transcription factor domain containing protein
LOC_Os02g12420.1	-1.178953105	-7.7848024	Receptor-like protein kinase precursor
LOC_Os12g10870	-1.20437027	-5.047916	Verticillium wilt disease resistance protein
LOC_Os05g12320	-1.225378218	-2.959296	Nodulin MtN3 family protein
LOC_Os08g41580	-1.377060129	-4.853546	Ubiquitin carboxyl-terminal hydrolase
LOC_Os07g33480.1	-1.413563692	-3.5746381	Cytochrome P450 domain containing protein
LOC_Os05g25400.1	-1.422277195	-20.296616	RNA binding protein, ribonuclease III activity
LOC_Os06g07020	-1.490540594	-2.8766186	ZOS6-01 - C2H2 zinc finger protein
LOC_Os11g47630.1	-1.517556133	-2.201833	ZOS11-10 - C2H2 zinc finger protein
LOC_Os11g36450	-1.736024455	-6.1	OsFBO15 - F-box and other domain containing protein
LOC_Os07g40240	-1.896348031	-5.9	GASR9- GASA/GAST/Snakin family protein

LOC_Os07g30980	-1.972235883	-4.069508	uvrD/REP Helicase family protein
LOC_Os01g02960.1	-2.410204319	-15.232705	Expressed protein
LOC_Os08g41590.1	-2.43270618	-6.2401733	Peptide transporter PTR2
LOC_Os01g74300.1	-2.515950627	-2.2411637	Metallothionein
LOC_Os05g25850.1	-2.762260832	-2.3229039	Superoxide dismutase
LOC_Os05g25350.1	-2.945038074	-22.431421	N/A
LOC_Os06g03770	-3.610416363	-2.0081406	ABC transporter
LOC_Os05g41760.1	-3.774301482	-2.0487518	AP2 domain containing protein
LOC_Os05g25650	-3.881953183	-51.846687	N/A
LOC_Os11g36200	-4.953760087	-70.49862	Receptor-like protein kinase 2
Rice OS.15501.1	-7.365138316	-27.63895	
LOC_Os03g26229	-10.71443933	-12.657807	
LOC_Os01g57270.1	-34.4835066	-10.562866	Disease resistance RPP13-like protein 1
LOC_Os11g36160	-44.75860868	-19.131111	Receptor-like protein kinase 2 precursor
LOC_Os06g06490	-81.01512963	-21.283022	U-box domain containing heat shock protein

Table S2. List of primers used in study

**A. List of primes used for validation of microarray by qRT-PCR**		
**S.No.**	**Gene Id**	**Forward primer (5' to 3')**
1	LOC_Os01g09320_F	GCCTCGGTGTTGTAATCTCTG
2	LOC_Os01g09320_R	GATCCCTTCTCAAAGTTCTCCTG
3	LOC_Os01g73000_F	TTCTTGAGAACGACAACGCTG
4	LOC_Os01g73000_R	GTTAAGCAGATTGGGCAAAC
5	LOC_Os07g40240_F	TTCCAGGATCTCACCGTCG
6	LOC_Os07g40240_R	CCACCTTCAGCGAGCAC
7	LOC_Os11g36450_F	GTGGCATTTGGTTATCAATCTGG
8	LOC_Os11g36450_R	CACTTGCATTGGGCCTCTT
9	LOC_Os10g39260_F	CGCCGTTCGTCAACATCTC
10	LOC_Os10g39260_R	AGCCCTTCGAGTTGGACCTT
11	LOC_Os02g43840_F	GGGTGAATCGGCTAGCAAGA
12	LOC_Os02g43840_R	ACGCCCAACAGGAGAAACCT
13	LOC_Os11g35220_F	CGGAATGGTGGACATGAAAAA
14	LOC_Os11g35220_R	GAACAATGACCCAAAAGTTTGATG
15	LOC_Os02g10780_F	TCTACAAGCTCGTGAAGGAATGC
16	LOC_Os02g10780_R	CCCCTTCGCTGTCATCTTTC
17	LOC_Os12g29950_F	TTCGTTGTGTCGGTGTTTGG
18	LOC_Os12g29950_R	GCGTAGTTCCAGATGCTGATGA
19	LOC_Os07g29770_F	CAACTATCGCACCTCCAAACCT
20	LOC_Os07g29770_R	CAAACACCGCAAGCTGATGT
21	LOC_Os03g24970_F	AGACATAGCAAAGTCAAAGGGAAGA
22	LOC_Os03g24970_R	TCGTTGACAATGACTGCAATGA
23	LOC_Os02g42690_F	ACTACACGAGCCCTTACCACATG

24	LOC_Os02g42690_R	GCGAACACGACAAGGAAGAAC
25	LOC_Os12g08220_F	TGTGTCTTTGGGAACATTGCA
26	LOC_Os12g08220_R	CGTTACCGTGGTGAACATCAA
27	LOC_Os11g36200_F	GGAGAGACATTGCGAGAAGGA
28	LOC_Os11g36200_R	ACAGTGAGAAGCAGGACAATGC
29	LOC_Os05g12320_F	ATGACTGTGTTTTTCTCAAGCTTTTC
30	LOC_Os05g12320_R	AGCCGTACATTGATATGGAAGACA
31	LOC_Os08g41580_F	CGCAGTTCCGAGGCATTG
32	LOC_Os08g41580_R	CCCTCCATTTCCTTTCATCCT
33	LOC_Os06g03770_F	TGCTGCACTGAAAACCCAAA
34	LOC_Os06g03770_R	CCGTGGATAAAGCCGAGCTA
35	LOC_Os07g30980_F	CTGGCTTTGATTCATTTCGTAGAG
36	LOC_Os07g30980_R	TGCCCGTGTAGAGCGATAGTT
37	LOC_Os12g10870_F	ATTGAGGACCCTTATGCTGATTG
38	LOC_Os12g10870_R	GTACAAGCCCCCCGACAGT
39	LOC_Os06g07020_F	AAGCGCCAGGTGAAGCATT
40	LOC_Os06g07020_R	GTGTCGGTGAAATCGGAGTCA
41	LOC_Os06g30920_F	ACCCCCTGTTCCTCATTTGTG
42	LOC_Os06g30920_R	CGTGGACGAGAATCGAATTATG
43	LOC_Os02g29774_F	CATCAGTGAAGTCTTTTCAGGTACTTG
44	LOC_Os02g29774_R	AAGTGAGATCATGTCGTGCAACA
45	LOC_Os05g06630_F	CGTCACGCTCTTCACTTACTTCA
46	LOC_Os05g06630_R	TATCAACCGTCCCTGCACAA
47	LOC_Os05g25650_F	GCCTGCGAAAACCGGATT
48	LOC_Os05g25650_R	CGCAAAATAAAGGAAACATACTGGTA
49	Rice OS.15501.1_F	AGCTGCTACTGTCCCTTCCTGTA
50	Rice OS.15501.1_R	GCGACAGATGCCTCTTTCCT
51	LOC_Os07g31884_F	TGCCGCTCTCCATCTTCTTC
52	LOC_Os07g31884_R	TGTCAGCCCGCAGATCAAA
53	LOC_Os07g29794_F	TTTGATGCTGGAGAACGAGAGA
54	LOC_Os07g29794_R	CGCCTTCTGCTCCTCATCAT
55	LOC_Os11g36160_F	TGCGTCATCGGAATCTTGTC
56	LOC_Os11g36160_R	CCGTTGGGCATGAAGTCATA
57	LOC_Os06g06490_F	CCAGCGACATCTCCTTCTTGA
58	LOC_Os06g06490_R	CCCTCACGCACTCCTGGTA
59	LOC_Os05g41540_F	GTGCCTGATAGCTTCGATGATTT
60	LOC_Os05g41540_R	GACGGACCAGGAGGATTGC
61	LOC_Os05g18650_F	CAAGGGCAATTTACAGCTTTGG
62	LOC_Os05g18650_R	TCCCTTGCCCCTCTCAGTT
63	LOC_Os03g26229_F	CCGCCTCCAGACCATTGT
64	LOC_Os03g26229_R	TGTGGCATTGCCTGATGAGT
65	LOC_Os01g02960_F	CTGTCCTCAGCGTCAACGAA
66	LOC_Os01g02960_R	CCAAGACTGTCCCCGAACTC
67	LOC_Os05g25350_F	CAAACTGCTCAGGCAGCAAA
68	LOC_Os05g25350_R	TCTGTGTGGATGGGAAAAAGG
69	LOC_Os06g35574_F	CACGGACAGCAAGCTCTTC

70	LOC_Os06g35574_R	TTAGTTGGCTCCACATGTCATC
71	LOC_Os03g29850_F	ACCGGCATCTTCATCTACGT
72	LOC_Os03g29850_R	AGGTGTCCCAGATCATGACG
73	LOC_Os03g29850_F	GAGGAGCTGGCGGATGTGT
74	LOC_Os03g29850_R	CCACAAACGCACAGGGAAGT
75	LOC_Os12g08270_F	TGCAGCAGGACCTCTTGAAA
76	LOC_Os12g08270_R	TCCATAATCGGCGACTGTCA
77	LOC_Os02g09220_F	GGTGGACATGATGGAGGAGT
78	LOC_Os02g09220_R	CGACATAGGCACACGAACAG
79	LOC_Os05g25400_F	AAGCCCCGTTCGCTCTACA
80	LOC_Os05g25400_R	TGCTCAGTGGTGGAAAGAAGAA
81	LOC_Os07g33480_F	CCCATCCTCCTCCACCTTGT
82	LOC_Os07g33480_R	ACGAATGGGAAGCCAAGTGA
83	LOC_Os05g25850_F	ACCTACGTCGCCAACTACAA
84	LOC_Os05g25850_R	TTGAACTTGATGGCGCTCTG
85	LOC_Os11g47630._F	CATGTGCGGCAAGGTGTT
86	LOC_Os11g47630_R	TGTGCCATTACGACTCACCA
87	LOC_Os09g39930_F	CAGGGACAACAACAGCAACC
88	LOC_Os09g39930_R	CTTGTGCCGGTAAGTCACTG
89	LOC_Os08g41590_F	CGGCCACCTCGACTACTTCTAC
90	LOC_Os08g41590_R	ACTGGTGTTTGATGGTACAAGCA
91	LOC_Os02g12420_F	GTTCTAATCAGCTGGCAGGC
92	LOC_Os02g12420_R	TTTGCAGCGATGGTATGCTC
93	LOC_Os01g74300_F	GATGCAAGTACTCTGAGGTGG
94	LOC_Os01g74300_R	CAGCACCAGACCCCTTG
95	LOC_Os01g57270_F	TGTCCAATTTGCGCCCTAAG
96	LOC_Os01g57270_R	TGGGTGCTGTGACCATAAGT
97	LOC_Os05g41760_F	ATGGAGCTGGACATGGGAG
98	LOC_Os05g41760_R	ACACCCGCGTCTTCTTCCA
99	LOC_Os03g50885_F	GAAGTACAGTGTCTGGATTGGAG
100	LOC_Os03g50885_R	CGTACTCAGCCTTGGCAATC
101	LOC_Os08g03290_F	GGAAAGCTCAAGGGAATCATAGG
102	LOC_Os08g03290_R	TTAAGAGCAATTCCAGCCTTGG
**B. List of primes of dehydration responsive genes**		
**S.No**	**Name**	**Primer 5' to 3'**
1	OsMYB2 RT_F	GGGCTGAAACGCACAGGCAAGA
2	OsMYB2 RT_R	CTGCTTGGCGTGCTTCTGC
3	J033099M14 RT_F	CTCAAATCAAGGCGTCAACTAAGA
4	J033099M14 RT_R	TTGTCAATATATACGTGGCATATACCA
5	J033031H21 RT_F	CGCCCCTCCCCGTATCT
6	J033031H21 RT_R	AGGAATGCGGCAACAAGTG
7	OsNHX1 RT_F	ACACGACCTCCGACTAC
8	OsNHX1 RT_R	TCATTGACCCAGCGATT
9	OsLEA RT_F	CGGCAGCGTCCTCCAAC
10	OsLEA RT_R	CGGTCATCCCCAGCGTG
11	OsDERB2A RT_F	GCTGCACATCAGCACCTTCA

12	OsDERB2A RT_R	TCCTGCACCTCAGGGACTAC
13	OsRAB16A RT_F	CACACCACAGCAAGAGCTAAGTG
14	OsRAB16A RT_R	TGGTGCTCCATCCTGCTTAAG
15	OsDREB1A RT_F	CATGGCCGGTGAACTTTGAC
16	OsDREB1A RT_R	CTCGTCGTCGTTCAGTCCAG
**C. List of primes of Phosphate starvation induce genes**		
1	OsSPX1 RT_F	GACCAGCTTCTACCATCAAACG
2	OsSPX1 RT_R	AGTTCCTGCTGCTCCTCTGG
3	OsPHO2 RT_F	GGCTATCGGAACTTATGG
4	OsPHO2 RT_R	AAGAAGGCAGAGGAGGTATC
5	OsPHR1 RT_F	CACAAGAAGGGAAAACTACCGATG
6	OsPHR1 RT_R	TCAAGATTCATGCACTCTACGACGC
7	OsPHR2 RT_F	CGCTTTGTAGATGCTGTCAATC
8	OsPHR2 RT_R	AGACCCTCATCACATCCTCATTATC
9	OsIPS2 RT_F	CCT TCTTCTGGATTCCTCTC
10	OsIPS2 RT_R	AGTTCACCACAAAAGATACAGTAG
11	OsSQD RT_F	CTGAAAACGGTAATGGATAGG
12	OsSQD RT_R	AACACCACCAGCACGAGC
13	OsPAP RT_F	ATACTGGCAGCCGACGGATGA
14	OsPAP RT_R	GAGGGAGCTGGAGCGGAGAA
**D. List of primes of nitrogen and ammonium transporter**		
**S.No**	**Name**	**Primer 5' to 3'**
1	OsNAR2.1 RT_F	CAAGGACAAGGCGTGCCAG
2	OsNAR2.1 RT_F	GCGATGGAGAAGGTGGAG
3	OsNRT1.1 RT_F	CGAGGTTGGTGCATTTTGTG
4	OsNRT1.1 RT_R	GCCGTGGTGTTCTCTTTTTTT
5	OsNRT1.2 RT_F	GCGGCGAGTCCCTGAG
6	OsNRT1.2 RT_R	CGACGGCGTAGATGAATGA
7	OsNRT2.1 RT_F	GCGACCGAGACCAGCAATAC
8	OsNRT2.1 RT_R	TTCATCACCGTTTGCAACAAG
9	OsNRT2.2 RT_F	GCCGGAGCACGCCTAAT
10	OsNRT2.2 RT_R	AAACGGTAACAAAACGTTCAACAG
11	OsAMT1.1RT_F	GGTCATCTTCGGGTGGGTCA
12	OsAMT1.1 RT_R	CGTGCCGTGTCAGGTCCAT
13	OsAMT1.2RT_F	GAAGCACATGCCGCAGAC
14	OsAMT1.2 RT_R	GACGCCCGACTTGAACAGC
15	OsAMT1.3RT_F	GCGAACGCGACGGACTA
16	OsAMT1.3RT_R	GACCTGTGGGACCTGCTTG
